# Outcomes for surgical procedures funded by the English health service but carried out in public versus independent hospitals: a database study

**DOI:** 10.1136/bmjqs-2021-013522

**Published:** 2021-09-07

**Authors:** Hannah Crothers, Adiba Liaqat, Katharine Reeves, Samuel I Watson, Suzy Gallier, Kamlesh Khunti, Paul Bird, Richard Lilford

**Affiliations:** 1 Health Informatics, University Hospitals Birmingham NHS Foundation Trust, Birmingham, UK; 2 Institute of Applied Health Research, University of Birmingham, Birmingham, UK; 3 Diabetes Research Centre, University of Leicester, Leicester, UK; 4 Institute for Translational Medicine, University Hospitals Birmingham NHS Foundation Trust, Birmingham, UK; 5 West Midlands Academic Health Science Network, Birmingham, UK

**Keywords:** patient safety, quality measurement, surgery

## Abstract

**Background:**

The outcomes of elective surgery in public versus Independent Sector Healthcare Providers (ISHPs) are a matter of policy relevance and theoretical interest.

**Methods:**

Retrospective study of all National Health Service (NHS) hospitals and ISHPs in England that provided NHS-funded elective surgery. We used data from the England-wide Hospital Episode Statistics to study 18 common surgical procedures performed between 2006 and 2019. In-hospital outcomes included length of stay, emergency transfers to another hospital or death. Posthospital outcomes included readmission or death within 28 days. Outcomes were compared for each operation type by propensity score matching and survival analysis.

**Results:**

The data set included 3 203 331 operations in 734 NHS hospitals and 468 259 operations in 274 ISHPs.

In-hospital outcomes: Across all 18 included operation types, length of stay was significantly longer for patients treated in NHS hospitals compared with ISHPs. Effect sizes ranged from a hazard ratio (HR) of 2.15 (95% CI 1.72 to 2.68) for total hip replacement to 1.07 (95% CI 1.05 to 1.09) for wisdom tooth removal; a mean difference of 2.49 and 0.02 days, respectively.

Postdischarge outcomes: Treatment at an ISHP was associated with a lower risk of emergency readmission compared with NHS treatment. HRs ranged from 0.36 (95% CI 0.28 to 0.46) for lumbar decompression to 0.75 (95% CI 0.67 to 0.85) for cholecystectomy; absolute risk differences of 1.5 and 1.3 percentage points. There was no difference in mortality.

**Conclusion:**

Elective surgery in an ISHP is associated with shorter lengths of stay and lower readmission rates than treatment in NHS hospitals across 18 operation types. The data were matched on observable covariates, but we cannot exclude selection bias due to unobserved confounders.

## Introduction

An Independent Sector Healthcare Provider (ISHP) is a private sector healthcare company that is contracted by the National Health Service (NHS) for the provision of healthcare.^1^ NHS spending on ISHPs, as a proportion of total NHS revenue spending, increased steadily from 3% in 2006/2007 to 7.5% in 2015/2016.^2^ Purchase of elective care from ISHPs by NHS hospitals is one of the fastest growing areas of expenditure on the independent sector.^3^ In 2009, the NHS constitution made it a right for patients in England referred for non-urgent hospital treatment to be able to choose to be treated by any provider (including an ISHP) listed in a national directory.^4^ The result is that people whose treatment is funded by one third-party payment system (the NHS) come to be treated by two different types of provider: public (NHS) and independent. The independent providers may be either for profit or not for profit. Reimbursement levels are the same irrespective of which type of organisation provides treatment, and providing data to NHS Digital is a prerequisite for reimbursement irrespective of provider type. This arrangement, where one commissioner funds treatment from more than one provider type, entails a ‘natural experiment’ for comparison of outcomes across different types of provider. Further details on the operation of the system are provided in online supplemental text 1.

10.1136/bmjqs-2021-013522.supp1 Supplementary data

Concerns have been raised about whether NHS patients treated by ISHPs receive the same standard of care as they would in an NHS hospital. The following factors have been highlighted as potential risks of elective surgery in ISHPs^5 6^: (A) the need for emergency transfer to an NHS hospital should complications arise in settings where intensive care is not provided; (B) working in an unfamiliar environment and having to use equipment with which the surgeon is unfamiliar; (C) putatively weaker governance arrangements and quality control processes; (D) frequent reliance on a single on-call resident medical officer to provide postoperative care; (E) permitting surgeons to exceed the NHS standard, whereby they should be available within 45 min having carried out an operation.

We found four research papers that have compared outcomes for NHS-funded patients undergoing specific types of surgical procedure in NHS hospitals compared with ISHPs (online supplemental table 1).^7–10^ These studies reported that case-mix adjusted outcomes, including persistent pain following joint replacement, postoperative complications, length of stay and quality of life, were better for NHS patients treated in private hospitals than in NHS hospitals. In addition, the Independent Healthcare Providers Network, which represents a wide range of ISHPs, has reported better performance for ISHPs when compared with NHS hospitals on overall patient satisfaction and, for hip and knee replacements, on patient-reported outcome measures.^11^ Our study has three main advantages over previous work. First, we cover a variety of operation types rather than being restricted to one or two classes of operations. Second, we cover an entire heath system (the whole of England). Third, we include operations over an extended period of nearly one and a half decades.

10.1136/bmjqs-2021-013522.supp2 Supplementary data

## Methods

Our study used data extracted from the Hospital Episode Statistics database (HES; NHS Digital) for April 2001 onwards, linked to mortality data from the Office for National Statistics (ONS). Socioeconomic deprivation was represented by Index of Multiple Deprivation (IMD) rankings for the patient’s residential address.^12^ This observational study was registered with the local Clinical Audit Department (Clinical Audit Registration and Management System number 15675). Data were used in line with the data sharing agreement with NHS Digital. The HES database comprises all episodes of care for patients who access care that is free at the point of use in England, including those treated at centres run by the independent sector.^13^ HES data include information on diagnoses (including comorbidities) and operations, patient demographics (such as age, sex, ethnicity and postcode) and administrative data (hospital provider, admission/discharge method, consultant specialty). The accuracy of the primary procedure and primary diagnosis fields in HES data is known to be high.^14^ Hospital episodes are linked by patients through derived HES ID values, which are matched to patient records based on NHS number and other patient identifiable details.^15^ This enables readmissions to be identified wherever they occur in England. We report our results in line with the Strengthening the Reporting of Observational Studies in Epidemiology criteria.^16^

### Data extraction and cleaning

The full list of operations carried out across both the NHS and the independent sector is very long and includes operations that are done infrequently or that carry minimal risk (eg, injections of therapeutic substances or diagnostic procedures). We therefore confined our attention to more commonly performed operations (a minimum of 4000 records in each provider-type group from the period 2001 to 2019) and those that had more than negligible risk. This selection preceded any analysis. The full list of operations that fulfilled the first criterion is given in online supplemental table 2. We retained 18 operations that satisfied both eligibility criteria (table 1).

10.1136/bmjqs-2021-013522.supp3 Supplementary data

**Table 1:** A list of operations passing both criteria for inclusion in this study

Operation codes (OPCS-4)	Specific operation type	Generic operation type
F091	Surgical removal of impacted wisdom tooth	Wisdom tooth
F093	Surgical removal of wisdom tooth NEC	
J183	Total cholecystectomy NEC	Cholecystectomy
M653	Endoscopic resection of prostate NEC	Prostate resection
Q074	Total abdominal hysterectomy NEC	Hysterectomy
T212	Repair of recurrent inguinal hernia using insert of prosthetic material	Inguinal hernia (IH) repair
T242	Repair of umbilical hernia using insert of prosthetic material	Umbilical hernia (UH) repair
T243	Repair of umbilical hernia using sutures	
T272	Repair of ventral hernia using insert of prosthetic material	Ventral hernia (VH) repair
V255	Primary posterior decompression of lumbar spinal cord NEC	Lumbar decompression
W371	Primary total prosthetic replacement of hip joint using cement	Total hip replacement (THR)
W381	Primary total prosthetic replacement of hip joint not using cement	
W391	Primary total prosthetic replacement of hip joint NEC	
W931	Primary hybrid prosthetic replacement of hip joint using cemented acetabular component	
W941	Primary hybrid prosthetic replacement of hip joint using cemented femoral component	
W401	Primary total prosthetic replacement of knee joint using cement	Total knee replacement (TKR)
W411	Primary total prosthetic replacement of knee joint not using cement	
W421	Primary total prosthetic replacement of knee joint NEC	

Frequency of these operation types is specified in table 2.

NEC, not elsewhere classified;OPCS-4, OPCS Classification of Interventions and Procedures version 4.

**Table 2:** Data set characteristics

	Count at ISHPs (% of group)	Count at NHS site (% of group)	Median length of stay (IQR), days, both groups	28-day readmission proportion (%), both groups*	
				Within specialty	All cause
Total inpatient operations	468 259	3 203 331	2 (0–4)	2.8	5.3
Median age (IQR)	64 (51–72)	57 (39–70)	NA	NA	NA
Sex					
Male	202 080 (43.2)	1 239 148 (38.7)	2 (0–3)	2.5	5.2
Female	266 179 (56.8)	1 964 183 (61.3)	2 (0–4)	3.1	5.3
Generic operation type					
Cholecystectomy	56 153 (12.0)	656 976 (20.5)	1 (0–1)	5.3	7.1
Hip replacement	142 451 (30.4)	518 755 (16.2)	4 (3–6)	1.8	5.5
Hysterectomy	13 676 (2.9)	310 517 (9.7)	3 (2–4)	5.9	8.4
IH repair	8783 (1.9)	46 919 (1.5)	0 (0–1)	2.8	4.6
Knee replacement	150 834 (32.2)	554 743 (17.3)	4 (3–6)	1.9	5.7
Lumbar decompression	13 770 (2.9)	59 163 (1.8)	1 (1–2)	2.0	4.0
Prostate resection	10 190 (2.2)	150 664 (4.7)	2 (2–3)	4.5	8.9
UH repair	31 270 (6.7)	177 216 (5.5)	0 (0–0)	2.8	4.0
VH repair	6322 (1.4)	40 759 (1.3)	0 (0–1)	4.6	6.5
Wisdom tooth	34 810 (7.4)	687 619 (21.5)	0 (0–0)	0.3	1.1
Year of operation					
2006	2746 (0.6)	135 542 (4.2)	3 (0–5)	2.8	5.5
2007	4393 (0.9)	210 329 (6.6)	3 (0–5)	2.7	5.2
2008	9332 (2.0)	221 976 (6.9)	2 (0–5)	2.6	5.1
2009	13 612 (2.9)	227 037 (7.1)	2 (0–5)	2.7	5.3
2010	23 050 (4.9)	240 486 (7.5)	2 (0–4)	2.7	5.2
2011	30 373 (6.5)	243 700 (7.6)	2 (0–4)	2.8	5.2
2012	33 960 (7.3)	249 360 (7.8)	2 (0–4)	2.8	5.2
2013	38 584 (8.2)	247 253 (7.7)	1 (0–4)	2.8	4.9
2014	45 340 (9.7)	259 501 (8.1)	1 (0–3)	2.8	5.1
2015	48 386 (10.3)	248 852 (7.8)	1 (0–3)	2.7	5.1
2016	49 082 (10.5)	239 920 (7.5)	1 (0–3)	2.8	5.2
2017	54 137 (11.6)	237 737 (7.4)	1 (0–3)	2.9	5.3
2018	56 443 (12.1)	223 751 (7.0)	1 (0–3)	3.1	5.7
2019	58 821 (12.6)	217 887 (6.8)	1 (0–3)	3.2	5.9
Index of Multiple Deprivation quintile					
1 (most deprived)	58 046 (12.4)	606 142 (18.9)	1 (0–3)	3.4	6.0
2	78 162 (16.7)	646 250 (20.2)	1 (0–4)	2.9	5.4
3	100 595 (21.5)	664 635 (20.7)	2 (0–4)	2.8	5.2
4	113 908 (24.3)	649 870 (20.3)	2 (0–4)	2.7	5.1
5 (least deprived)	116 154 (24.8)	607 432 (19.0)	2 (0–4)	2.5	4.9
Unknown	1394 (0.3)	29 002 (0.9)	3 (0–5)	1.8	3.1
Charlson Comorbidity Index					
0	358 809 (76.6)	2 314 090 (72.2)	1 (0–3)	2.6	4.5
1–3	24 130 (5.2)	156 869 (4.9)	3 (1–5)	3.3	6.7
4+	85 320 (18.2)	732 372 (22.9)	3 (1–5)	3.6	7.6
Ethnicity					
White	348 850 (74.5)	2 566 935 (88.1)	2 (0–4)	3.0	5.6
Asian	8336 (1.8)	119 653 (3.7)	1 (0–3)	3.5	6.0
Black	2669 (0.6)	71 840 (2.2)	0 (0–3)	3.0	5.4
Mixed	1579 (0.3)	21 203 (0.7)	0 (0–2)	2.6	4.3
Other/unknown	106 825 (22.8)	423 700 (13.2)	1 (0–3)	1.7	3.4

Similar operation types have been grouped together in order to summarise the data.

*The percentage is calculated based on patients discharged alive (and not transferred to another hospital provider) within 60 days of their index admission.

IH, inguinal hernia; ISHP, Independent Sector Healthcare Provider; NA, not applicable; NHS, National Health Service; UH, umbilical hernia; VH, ventral hernia.

Our data comprised all spells of care that began between 1 April 2006 and 31 December 2019, which contained one of the included operations and that were the first operation of its type for the patient. By taking 1 April 2006 as the start of our study we were able to use at least 5 years of data (from 1 April 2001 onwards) to check whether a patient had undergone a prior operation of the same type. Following this, an operation was included only if it was listed as the primary operation undergone during the inpatient spell and if it took place as part of a non-emergency admission. Further data cleaning involved removing records with missing primary diagnosis information, records with unknown age, sex or provider type and any cases where patients were recorded as having the same operation on the same day as part of separate inpatient spells. The cohort selection process is shown in online supplemental figure 1.

10.1136/bmjqs-2021-013522.supp4 Supplementary data

### Calculation of derived variables

Patient residential address was matched to a quintile of the IMD using the following IMD versions^12^: IMD v2004 for activity within the 2006–2007 financial year, IMD v2007 for activity between 2007–2008 and 2009–2010, IMD v2010 for activity between 2010–2011 and 2013–2014, IMD v2015 for activity from 2014–2015 to present.

All patient diagnoses (up to 24) recorded during the index spell were used to calculate the Charlson Comorbidity Index.^17^ The presence of Elixhauser comorbidities was calculated based on all diagnoses from the first episode of the index spell and any spells occurring in the year prior to the index admission. International Classification of Diseases 10th Revision (ICD-10) codes for Elixhauser comorbidities were taken from Quan *et al*,^18^ with the addition of a category for dementia (ICD-10 F00–F03 and F05.1). Length of stay was calculated as the difference between the operation date and discharge date. We defined within-specialty readmissions as an emergency inpatient admission coded within the same specialty as the operation (see online supplemental table 3 for specialties relevant to each operation type). For all-cause readmissions, the specialty requirement was removed. For both categories of readmissions, the readmission date had to follow the initial admission date and take place within 28 days of the discharge date. The readmission could be to the same or a different hospital (of either provider type) as the initial admission. Data on in-hospital deaths (during the index admission) were obtained from the HES database, whereas data on deaths following discharge were obtained via linkage to death records from the ONS.

10.1136/bmjqs-2021-013522.supp5 Supplementary data

### Propensity score matching

For each operation type, propensity score matching was used to create comparable control (NHS) and treatment (ISHP) groups. We implemented the nearest neighbour method of propensity score matching in R using the package ‘MatchIt’,^19^ specifying a calliper of 0.1. Propensity scores were calculated using a logistic regression model. Categorical variables included in the model were sex, ethnicity (including ‘unknown’), IMD quintile (including ‘unknown’) and operation year; while age and Charlson Comorbidity Score were included as (linear) continuous variables. All variables were determined from data recorded at the time of admission for the operation of interest. Since there were many more operations in NHS hospitals than ISHPs for all operation types, no ISHP records were excluded by the matching algorithm—one NHS record was chosen for every ISHP record and no ‘pruning’ was required as all matches were within the prespecified matching threshold. However, the matching process was computationally expensive; therefore, for operation types where more than 20 000 operations had taken place in ISHPs, the whole data set for that operation type was randomly subsampled prior to matching.

To validate the quality of the matching, the standardised mean difference was calculated before and after the propensity score matching. We judged standardised mean difference values greater than 0.1 to indicate significant imbalance between matched groups with regard to the covariate under consideration. Additionally, we visualised the effect of propensity score matching by plotting histograms of propensity scores in the NHS hospital versus ISHP groups before and after matching.

### Statistical analyses

#### Grouping outcomes

**Figure 1:**
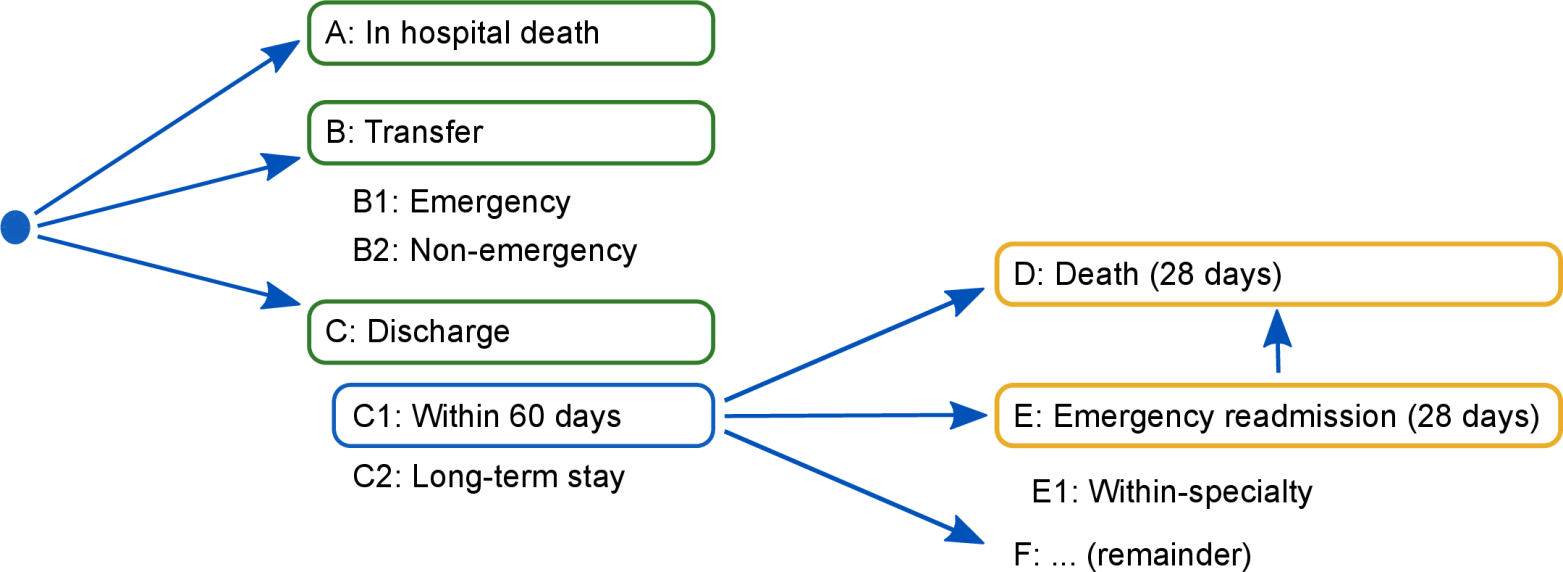
Diagram showing possible outcomes. Outcomes included in the in-hospital survival analyses are highlighted with a green box. Outcomes included in the postdischarge survival analyses are highlighted with an orange box. The blue box indicates that only patients discharged from hospital within 60 days are included in the postdischarge analyses. Only 65 patients (0.01%) remained in hospital beyond 60 days (category C2; table 3).

In figure 1, possible outcomes are represented diagrammatically according to where they come in the patient pathway. On the basis of the logic represented in the figure, outcomes are classified into two groups: ‘in-hospital outcomes’ and ‘outcomes with 28 days post discharge’.

#### In-hospital outcomes

We used survival analysis methods to analyse the time from operation to one of three competing outcomes that terminated a spell of care: discharge, death or transfer to another hospital. We further split transfers into emergency or non-emergency transfer according to how they had been coded by the receiving hospital. We graphically examined the cumulative incidence of each outcome with respect to time postoperation, allowing for competing risks using a multistate model formulation in the R ‘survival’ package.^20^ We estimated adjusted hazard ratios (HRs) for each outcome by fitting a Cox proportional hazards model for each outcome separately, with provider type as the sole predictor, right censoring if a different outcome occurred before the outcome of analysis and accounting for clustering within treatment provider sites.

#### Outcomes within 28 days postdischarge

The approach for examining differences in postdischarge events was similar. Here, emergency readmission (either within specialty or all cause) and death were the events under consideration, and all patients were censored at 28 days after discharge if they had not experienced an event by this point. When fitting Cox proportional hazards models for death within 28 days, we considered all deaths regardless of whether they were preceded by an emergency readmission. The ‘denominator’ population, that is, those used in the postdischarge analyses, was considered to be all patients who were discharged alive within 60 days of their index admission, without having been transferred to another hospital provider since their operation.

### Subgroup analysis

#### NHS versus for-profit hospitals

We repeated the propensity score matching and statistical analyses, restricting the data to only episodes of care in NHS hospitals or for-profit ISHPs to compare outcomes between these two provider types. The breakdown of ISHPs into not-for-profit and for-profit categories is given in online supplemental table 4 for all ISHPs contributing at least 10 operations to the data set.

10.1136/bmjqs-2021-013522.supp6 Supplementary data

#### Time epochs

Given the increasing use of ISHPs as a proportion of all NHS-funded operations, we tested the stability of our findings over time by examining cumulative incidence curves, stratified by time period within which the operation occurred (with ‘early’ defined as prior to 1 January 2014 and ‘late’ defined as 1 January 2014 onwards).

#### Interactions between place of surgery and propensity score

We examined heterogeneous effects of provider type by estimating a proportional hazards model that included provider type interacted with quartile of the propensity score.^21^ We used a χ^2^ test to test the null of no difference in treatment effects between propensity score quartiles against the alternative hypothesis of at least one quartile being different from the rest.^22^

### Sensitivity analyses

To check whether our results were sensitive to the choice of comorbidity index, we repeated the main survival analyses on a matched data set, obtained by including binary variables for each of the Elixhauser comorbidity categories in the logistic regression rather than the Charlson score.

Lastly, to examine whether our results were sensitive to the choice of propensity score matching as a method for removing bias, we repeated the main survival analyses on the raw data and instead adjusted for all the covariates (sex, ethnicity, IMD quintile, operation year, age and Charlson Comorbidity Score) within the Cox proportional hazards models in order to obtain adjusted HRs.

## Results

### Data set

We analysed 3 671 590 operations taking place between 1 April 2006 and 31 December 2019 from 274 ISHP sites and 734 NHS hospitals. Characteristics of the data set are shown in table 2. The proportion of operations taking place at ISHPs has increased over time from 2% in 2006 to 27% in 2019. A large majority (393 086; 84%) of operations that took place in ISHPs were in for-profit organisations (online supplemental table 4). Patients undergoing an operation at ISHPs were on average older, but with lower levels of comorbidity; lived in more affluent areas; and were more predominantly White (or had no ethnicity recorded), compared with those treated in NHS hospitals. These observations on patient demographics were confirmed by our propensity score model (online supplemental table 5).

10.1136/bmjqs-2021-013522.supp7 Supplementary data

### Matching

Online supplemental table 6 shows standardised mean differences between the two provider-type groups calculated for the factors sex, ethnicity, age, operation year, Charlson Comorbidity Index and deprivation. Prior to matching, the standardised mean difference was >0.1 for the majority of operation types for all factors except sex, indicating a noticeable imbalance between provider types for each of these factors. In the matched samples, no standardised mean difference exceeded 0.1. The matched groups were well balanced with respect to all covariates included in the model, as illustrated by the histograms of propensity scores before and after matching (online supplemental figure 2).

10.1136/bmjqs-2021-013522.supp8 Supplementary data

10.1136/bmjqs-2021-013522.supp9 Supplementary data

### Frequency of outcomes

The following results all compare the matched samples. Table 3 reports the number of events occurring for all 18 operation types combined and across the outcome types represented in figure 1. Event counts for individual operation types are included in online supplemental table 7. More than 99.5% of patients were discharged alive within 60 days of admission. In-hospital death and 28-day emergency readmission occurred more frequently for operations undergone in NHS hospitals, whereas emergency transfer to another hospital and death within 28 days of discharge (or within 28 days of transfer) occurred more frequently following operations in ISHPs.

10.1136/bmjqs-2021-013522.supp10 Supplementary data

**Table 3:** Event counts in the matched data

Event type	Count		% of total		% of discharged	
	NHS	ISHP	NHS	ISHP	NHS	ISHP
In-hospital death (A)*	92	9	0.04	0.00		
Emergency transfer (B1)*	180	216	0.07	0.09		
Non-emergency transfer (B2)*	1132	106	0.47	0.04		
Death within 28 days of transfer	10	11	0.00	0.00		
Long-term stay† (C2)*	53	12	0.02	0.00		
Discharge (C1)*	239 530	240 644	99.40	99.86		
Death‡ (D)*	87	109	0.04	0.05	0.04	0.05
Emergency readmission‡ (E)*	12 048	8073	5.00	3.35	5.03	3.34
of which within specialty	6334	3569	2.63	1.48	2.64	1.48
Remainder‡ (F)*	227 437	232 509	94.38	96.48	94.95	96.62
Total operations§	240 987	240 987				

*See figure 1 for label correspondence.

†Defined as >60 days in hospital.

‡D–E are events occurring within 28 days of discharge while F is the number of discharged patients who do not meet D or E within 28 days.

§Note that since these event counts are for the data after propensity score matching, the total count is much lower than for the raw data in table 2.

ISHP, Independent Sector Healthcare Provider; NHS, National Health Service.

### In-hospital outcomes

**Figure 2:**
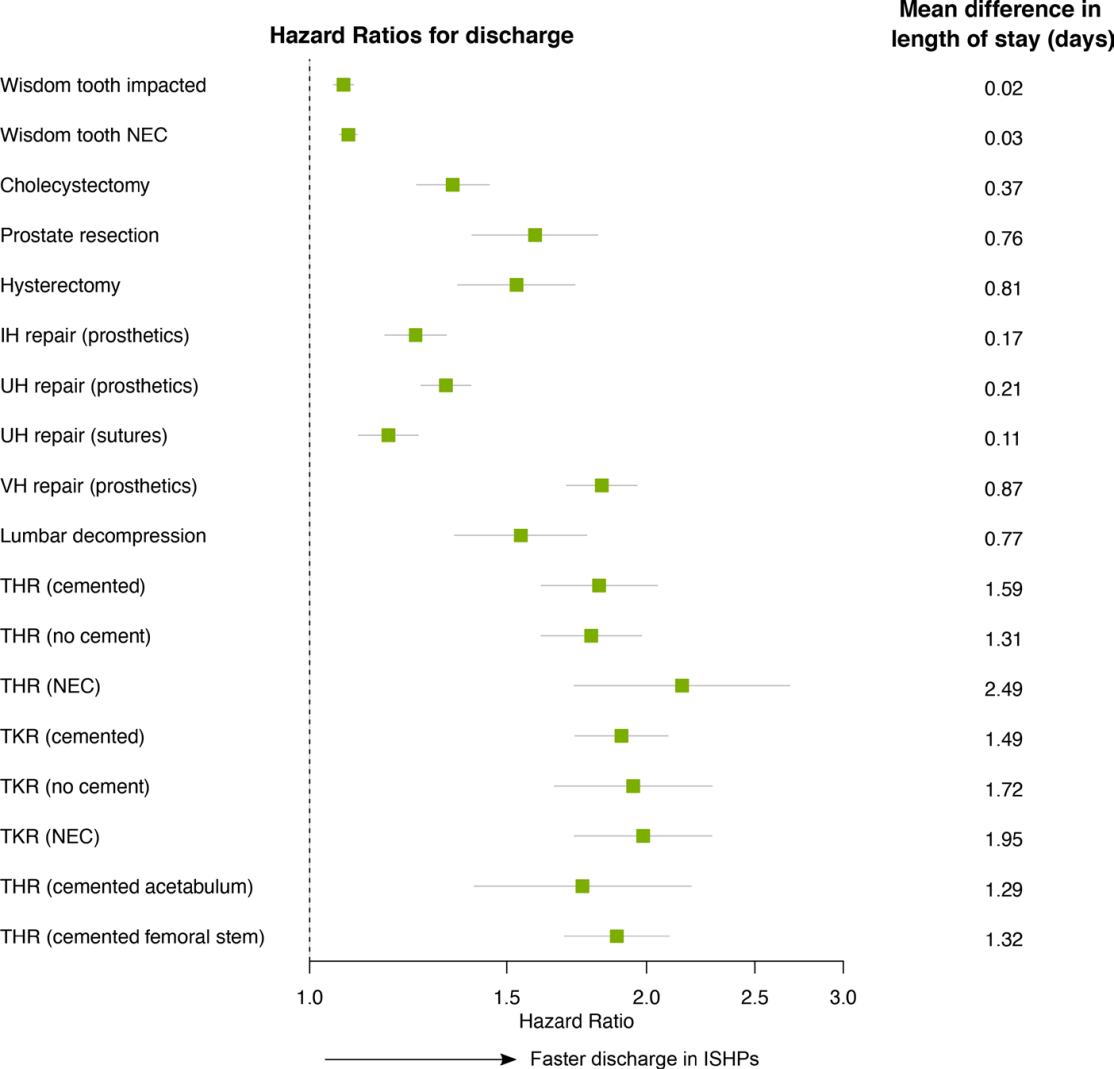
HRs and 95% CIs for the effect of provider type on time to discharge. Unadjusted Cox regression was used, with clustering of operations within hospital sites accounted for in order to calculate robust SEs. An HR greater than 1 indicates shorter times from operation to discharge in Independent Sector Healthcare Providers (ISHP). On the right-hand side, the corresponding mean difference in length of stay is given. This is defined as the mean length of stay for patients treated in National Health Service (NHS) hospitals minus the mean length of stay for patients treated at ISHPs. The means are calculated based on patients discharged alive within 60 days (category C1 in figure 1). IH, inguinal hernia; NEC, not elsewhere classified; THR, total hip replacement; TKR, total knee replacement; UH, umbilical hernia; VH, ventral hernia.

**Figure 3:**
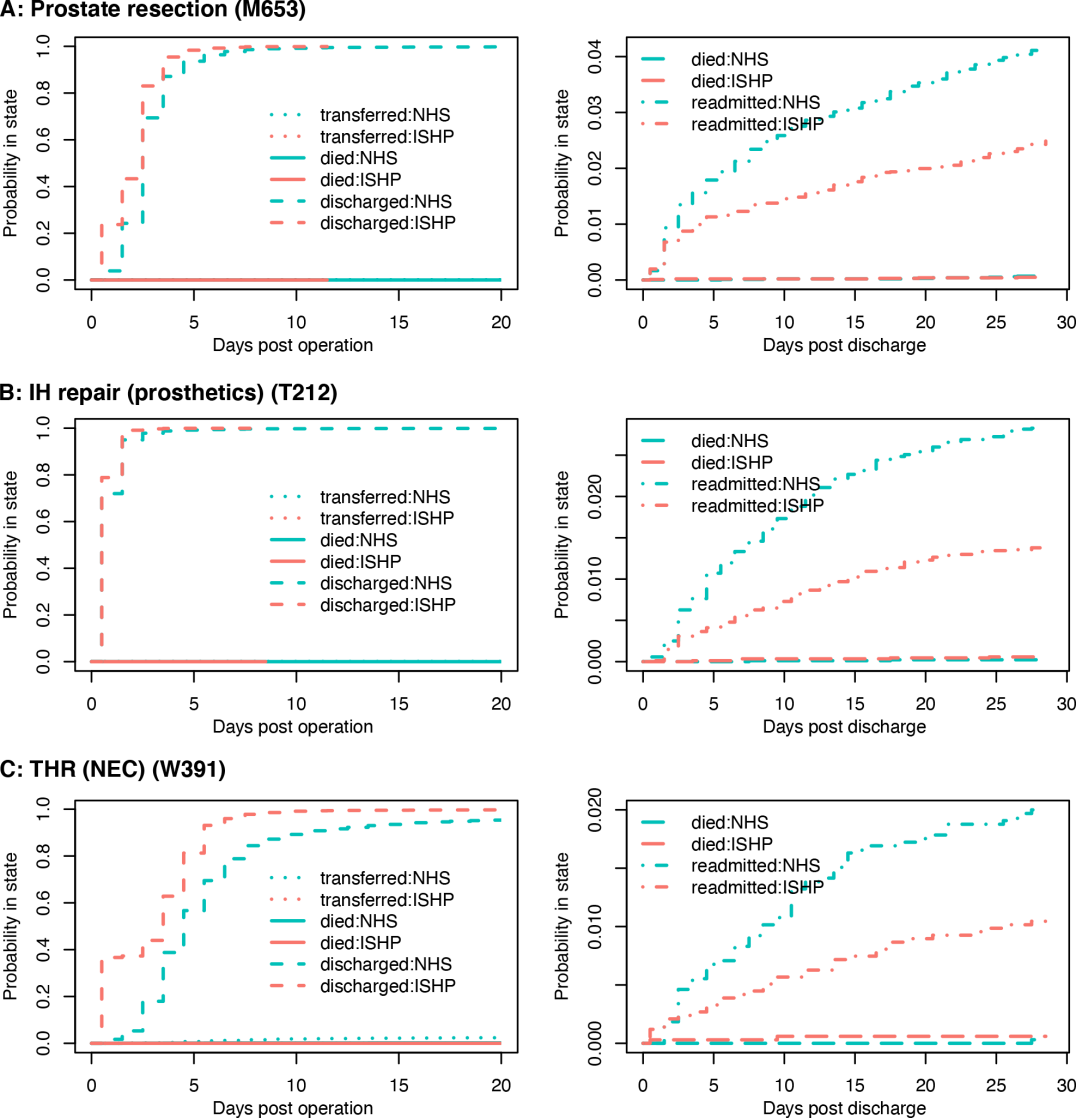
Cumulative incidences of all in-hospital and postdischarge events. Part A shows data for endoscopic resection of prostate NEC; Part B shows data for repair of recurrent inguinal hernia using insert of prosthetic material; Part C shows data for primary total prosthetic replacement of hip joint NEC. Left-hand side: cumulative incidence of all in-hospital events after operation, split by provider type (NHS hospital=blue; ISHP=red) and event type (between hospital transfers=dotted; in-hospital death=solid; discharge=dashed). Events are considered as competing risks, formulated as a multistate model. Right-hand side: similar plots for postdischarge events (death=long dashed; within-specialty emergency readmission=dot dashed). The three operation types shown here were chosen so as to best represent together all 18 sets of results (the full set is shown in online supplemental figure 3). IH, inguinal hernia; ISHP, Independent Sector Healthcare Provider; NEC, not elsewhere classified; NHS, National Health Service; THR, total hip replacement.

For all 18 types of operation, treatment in ISHPs was associated with shorter lengths of stay than treatment in NHS hospitals, resulting in HRs for discharge greater than 1, as shown in figure 2. Patterns of earlier discharge at ISHPs are also visible in the plots of cumulative incidence of in-hospital outcomes (figure 3 and online supplemental figure 3; left-hand panels). This difference was smallest in magnitude for wisdom tooth operations, cholecystectomy and inguinal or umbilical hernia repair, all of which had an HR for discharge and upper CI limit <1.5 (online supplemental table 8), as well as a mean difference in length of stay <0.4 days (figure 2). The difference was greatest in magnitude for hip and knee replacements, with HRs varying from 1.75 (95% CI 1.40 to 2.19) to 2.15 (95% CI 1.72 to 2.68), and mean differences in length of stay varying from 1.3 to 1.5 days across specific hip and knee operation types. For some types of hip and knee replacement the practice of discharging patients on the same day was a regular occurrence at ISHPs, while remaining very rare at NHS hospitals (online supplemental figure 3). For example, for total hip replacement (THR) (not elsewhere classified, NEC) nearly 40% of patients in our matched data set treated at ISHPs were discharged on the same day, compared with <5% of patients treated at NHS hospitals (figure 3C). The mean length of stay in the NHS-treated subsample matched to those discharged on the same day from ISHPs was approximately 5 days, which was comparable to the mean length of stay for all NHS patients undergoing this operation type.

10.1136/bmjqs-2021-013522.supp11 Supplementary data

10.1136/bmjqs-2021-013522.supp12 Supplementary data

The incidence of other in-hospital events (transfers and deaths) is very low in both groups of hospitals (table 3). Emergency transfers were more common after treatment in an ISHP than in an NHS hospital for seven of the 18 operation types, and in no case was the opposite effect observed. However, when it came to non-emergency transfers, the overall effect was reversed with more transfers following NHS than ISHP treatment for eight of the 18 operation types. There was only one operation where death rates differed and this was in favour of ISHPs. These data are shown in online supplemental table 8.

### Outcomes within 28 days postdischarge

**Figure 4:**
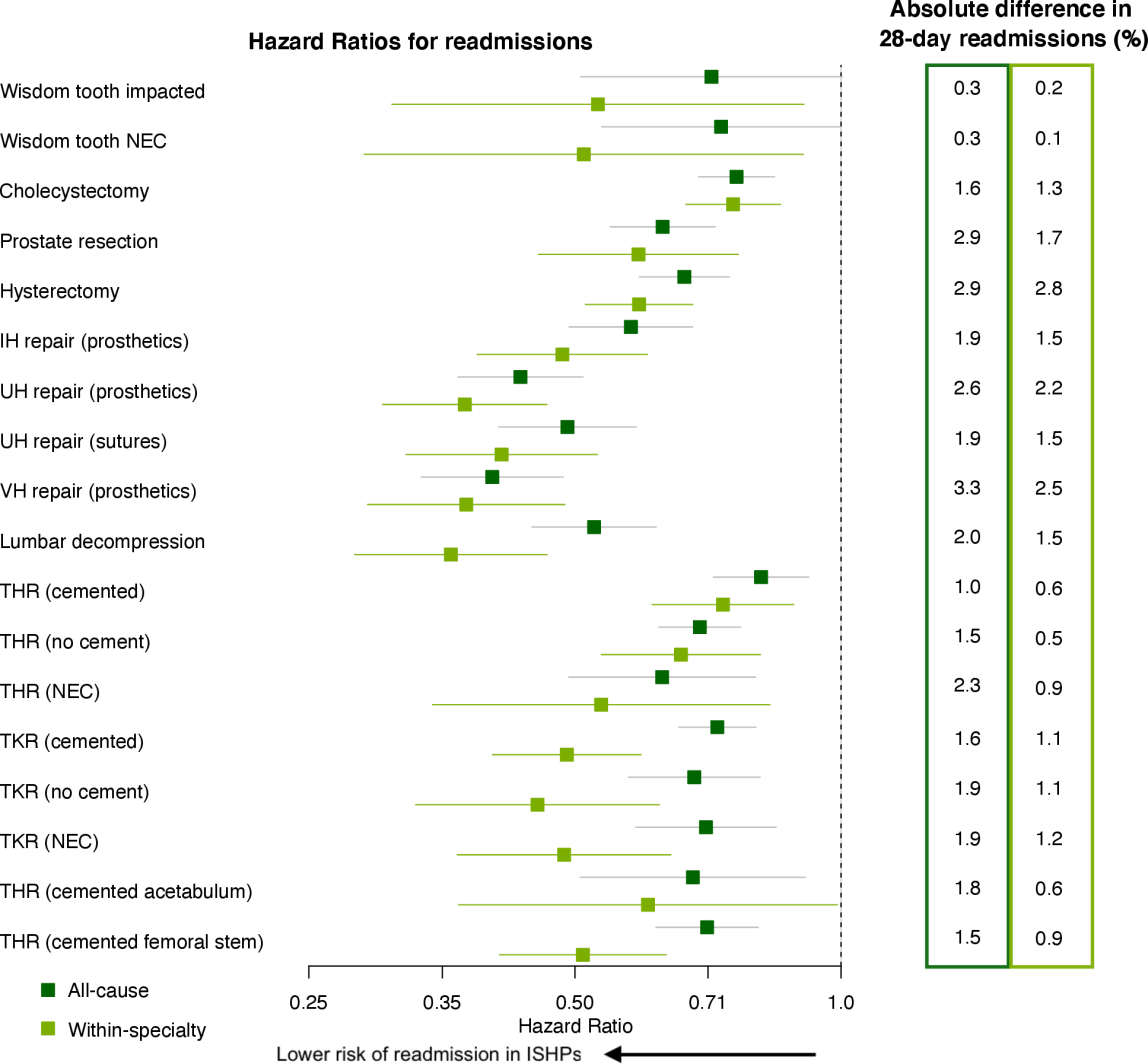
HRs and 95% CIs for the effect of provider type on time to emergency readmission. Unadjusted Cox regression was used, with clustering of operations within hospital sites accounted for in order to calculate robust SEs. Within-specialty admissions are shown in light green while all-cause readmissions are shown in dark green. An HR less than 1 indicates a lower risk of such a readmission for patients treated in Independent Sector Healthcare Providers (ISHP). On the right-hand side, the corresponding absolute difference in the number of patients readmitted within 28 days of discharge is given (category E in figure 1), as a percentage of the total number of patients discharged alive within 60 days of their operation (category C1 in figure 1). Since the number of 28-day emergency readmissions is always (for all 18 operations) greatest for patients treated at National Health Service (NHS) hospitals, this number represents the additional readmissions among patients treated at NHS hospitals, per 100 patients. The left-hand column is for all-cause readmissions while the right-hand column is for within-specialty readmissions. IH, inguinal hernia; NEC, not elsewhere classified; THR, total hip replacement; TKR, total knee replacement; UH, umbilical hernia; VH, ventral hernia.

Figure 4 shows the adjusted HRs for emergency readmission for outcomes up to 28 days following discharge. For all operation types the hazard for within-specialty readmission is significantly lower for operations taking place at ISHPs than for those taking place at NHS hospitals. The same holds for all-cause readmission, with the exception of impacted wisdom tooth removal where there is little to no evidence of a difference (online supplemental table 9). For some operation types the incidence of within-specialty readmission was similar between providers in the first 2–3 days following discharge, but for all 18 operation types the cumulative incidence showed a clear divergence after this point (figure 3 and online supplemental figure 3; right-hand panels). There were three operation types for which there was strong evidence that HR for within-specialty readmission was less than 0.5: umbilical hernia repair using insert of prosthetic material, ventral hernia repair and lumbar decompression (online supplemental table 9). For these three operations the absolute difference in readmissions within 28 days of discharge varied from 1.5 to 2.5 percentage points (figure 4). The smallest effect sizes were 0.74 (0.61–0.88) and 0.75 (0.67–0.85) for THR using cement and cholecystectomy, respectively. The corresponding absolute differences in readmissions within 28 days of discharge were 0.6 and 1.3 percentage points, respectively. Effect sizes were somewhat smaller when all-cause rather than within-specialty readmissions were considered. For all operation types there was no evidence of a difference between the risks of death in patients discharged from ISHPs versus NHS hospitals (online supplemental table 9).

10.1136/bmjqs-2021-013522.supp13 Supplementary data

### Subgroups: for-profit ISHPs, early versus late epoch and propensity score quartiles

When restricting the comparison to for-profit ISHPs and NHS hospitals we found that the direction and magnitude of effects were qualitatively very similar to the original comparison with all ISHPs (online supplemental figure 4 and online supplemental tables 10 and 11).

10.1136/bmjqs-2021-013522.supp14 Supplementary data

10.1136/bmjqs-2021-013522.supp15 Supplementary data

10.1136/bmjqs-2021-013522.supp16 Supplementary data

We stratified the data into two time windows: early (2006–2013) and late (2014–2019). Overall, qualitatively similar patterns in time to discharge and time to emergency within-specialty readmission were observed in both two time windows (online supplemental figure 5). However, the practice of same-day discharge for hip and knee replacements was only identifiable in ISHPs in the early time period data; patterns of time to discharge were more similar between ISHPs and NHS hospitals in the late period data. Turning our attention to within-specialty readmissions, there were some operations for which the magnitude of the effect of provider type was smaller in the late time period data (such as THR not using cement and hybrid hip replacement using cemented femoral component), and some for which the magnitude was larger in the late time period data (such as prostate resection and THR using cement).

10.1136/bmjqs-2021-013522.supp17 Supplementary data

We examined the heterogeneity of the effect of provider type with respect to the propensity of being treated in an ISHP (online supplemental figure 6). In other words, we looked to see whether, in a model with an interaction between provider type and propensity score, the estimated HR varied by propensity score quartile. The overall picture was that HRs for all outcomes were similar across the four quartiles of the propensity score within an operation type. When we examined the outcomes and operation types for which HRs did noticeably vary by propensity score quartile, we found no consistent pattern. For example, where there was evidence of an interaction between provider type and propensity score quartile, there were roughly equal numbers of operation types for which the effect of provider type increased versus decreased in magnitude with increasing propensity score. Further details are provided in online supplemental figure 6.

10.1136/bmjqs-2021-013522.supp18 Supplementary data

### Sensitivity analyses

When using the Elixhauser comorbidity categories instead of Charlson score as part of the matching algorithm, we found there was little change to the estimated HRs for in-hospital or postdischarge outcomes (online supplemental tables 12 and 13).

10.1136/bmjqs-2021-013522.supp19 Supplementary data

10.1136/bmjqs-2021-013522.supp20 Supplementary data

Lastly, we obtained HRs for provider type from survival analyses performed on the raw (ie, unmatched) data, adjusting for the same list of covariates that had been used in the original matching algorithm. The resulting effect estimates were again qualitatively similar to the original estimates, but in this case the magnitude was, in general, somewhat increased (online supplemental tables 14 and 15). The larger number of records in the models meant it was possible to estimate HRs for rare events, such as death, for a few more of the operation types. However, CIs were still large. There was some evidence that the hazard of death in hospital was lower for some hip and knee replacement types when they took place in ISHPs (THR cemented: HR 0.2, 95% CI 0.1 to 0.4; THR no cement: HR 0.2, 95% CI 0.0 to 0.7; total knee replacement (TKR) cemented: HR 0.2, 95% CI 0.1 to 0.4), but for other hip and knee replacement types death within 28 days of discharge had a higher hazard at ISHPs (TKR NEC: HR 3.0, 95% CI 1.4 to 6.5; THR cemented acetabulum: HR 4.7, 95% CI 1.8 to 12.7).

10.1136/bmjqs-2021-013522.supp21 Supplementary data

10.1136/bmjqs-2021-013522.supp22 Supplementary data

## Discussion

### Main finding

Our analysis suggests that, across the range of operation types studied, patients treated in ISHPs are more likely to be discharged from hospital sooner (figure 2) and are less likely to be readmitted (figure 4). These findings are consistent across all 18 operation types, and the effect sizes in many instances are large. For instance, the risk of within-specialty readmission after lumbar decompression in an ISHP is about a third of that in an NHS hospital, changing to about a half when all-cause readmissions are considered. There is evidence that patients in ISHPs were more likely to be transferred to another hospital as an emergency, while in-hospital death and non-emergency transfer were more likely for patients treated in NHS hospitals.

### Strengths and limitations

This study includes over three and a half million operations, covers an entire country, and many operation types. However, precise as our study may be, it has two main limitations. First, the study is observational, so by itself it cannot prove that the better outcomes in ISHP hospitals were causal. Second, we were able to evaluate only a limited selection of outcomes. We now discuss these two issues.

### Selection effects

A number of factors determine whether a patient will have their operation in an independent facility. These are largely operational factors related to capacity in the NHS, but patient and clinician choice also plays a part (online supplemental text 1), opening the door for selection effects. We adjusted for differences in the observable characteristics of patients treated in ISHPs versus NHS hospitals by creating matched cohorts with respect to age, sex, deprivation, ethnicity and comorbidity score, as well as year of operation. However, there are differences in case complexity that are not captured by (fairly granular) ICD-10 codes recorded in HES data. Additionally, calculation of the comorbidity score relies on secondary diagnosis coding, which is more variable than primary diagnosis coding,^23^ and may be less complete in ISHPs than NHS hospitals.^24^ A selection mechanism might lead to a correlation between risk and probability of being treated in an ISHP. This may translate into heterogeneity of ‘treatment’ effect with respect to the propensity score.^25^ We did not find evidence of such an effect, but this does not disprove the possibility of residual confounding.

### Outcome measures

The range of outcome measures that we observed was limited by those available in the data set to which we had access. Our results do, however, complement the study quoted earlier,^11^ showing improved satisfaction among NHS-funded patients treated in ISHPs compared with NHS hospitals. As routine data sets mature we will be able to evaluate more, and arguably better, outcomes such as patient-reported outcome measures. For example, a recent cohort study compared the generic quality of life following hip replacement where the outcomes were slightly in favour of independent providers.^7^

### Possible causal mechanisms

In so far as outcomes may be causal, we speculate that length of stay and readmissions may be influenced by different causal mechanisms—operational efficiency with respect to length of stay and technical competence with respect to readmission. Regarding length of stay, time of discharge is influenced by the patient’s condition and by hospital policy and operational factors. For example, independent providers might have more incentives to implement same-day discharge policies, and there is evidence in the data that some ISHPs followed a policy, perhaps fashion, for day-case hip surgery for a period of time. Regarding readmission, we cited a number of reasons why outcomes may be worse in ISHPs, such as the effect of operating in a less familiar environment. However, it is also the case that admission rights to ISHPs are usually restricted to registered specialists, while less experienced trainees provide a high proportion of care in the NHS. We can thus speculate that greater technical efficiency may explain some of the findings in our study.

The finding that emergency hospital transfers were more common in ISHPs does not necessarily reflect on the safety of care. Many ISHPs lack the full range of services, including intensive care, required for management of an emergency case. Thus, given an emergency, a patient in an ISHP is more likely to be transferred than an equivalent patient in the NHS who is already likely to be in the institution of last resort.

### Interpretation

As stated in the Introduction section, plausible concerns have been expressed regarding the safety of elective surgery in the independent sector. Taken in the round, our findings provide a measure of reassurance that Independent Sector Healthcare Providers are providing an acceptable service. In the context of meeting the backlog of cases following the COVID-19 pandemic this may be a useful finding. But our results stop short of total reassurance, and ongoing scrutiny of a richer set of outcomes and further investigation of practice is required in both the NHS and ISHPs.

## Data Availability

Data may be obtained from a third party and are not publicly available. Linked HES and ONS data may be obtained from NHS Digital via the Data Access Request Service (DARS) and are not publicly available.
