# Supplementary material for: Outcomes for surgical procedures funded by the English health service but carried out in public versus independent hospitals: a database study
Source: BMJ Qual Saf. 2021 Sep 7;31(7):515–25. doi: 10.1136/bmjqs-2021-013522 (PMC9234423; doi:10.1136/bmjqs-2021-013522)
Supplement: Supplementary data [file bmjqs-2021-013522supp001.pdf]

SUPPLEMANTARY TEXT 1

Policy Context
The period covered by this study coincides with the promotion of patient choice as a policy initiative within the NHS. From 2006 patients referred to a specialist were to be given a choice of NHS providers, and from 2008 this was widened to allow patients to choose any provider listed in a national directory which included independent hospitals. From 2009 this was enshrined as a right for patients within the NHS Constitution,[1] and again promoted by the Health and Social Care Act of 2012.[2]

One of the reasons for promoting patient choice was because long waiting times existed for inpatient procedures and providers had little stimulus to change behaviour. It was hoped that by enabling patients to move more freely around a 'quasi market' [3,4] this would force providers to reduce waiting times and improve quality.

However, the concept of 'choice' presented in a number of different ways. Policy makers intended that choice should be a full range of public and independent providers of treatment. However, choice of outpatient appointment, which is the gateway to secondary care, relied on a provider having available outpatient capacity and appointment slots which were visible on the electronic booking system to referring GPs. If no capacity was available, then the provider would not be an option for the patient. Patients referred for high demand specialties (such as plastic surgery and neurosurgery) with long waiting times which the choice initiative aimed to stimulate shorter waiting times for, often received the most limited range of options.

Many patients who were offered choice did not act upon it, and so selected their local provider. There are few studies which have looked at how often choice was offered and acted upon. However, a survey by the Kings Fund [5] in 2009 found that 49% of patients were offered a choice of provider by their GP and of these 8% recalled being offered a private provider. Many patients who were offered more than one NHS provider felt they had been given choice, even if this did not include the option of an independent provider, even though this was doubtless what policy makers had intended.

There were organisational limitations on the types of procedure available through patient choice too. For instance, many complex or higher risk procedures could not be offered in many private hospitals. Private providers also had to deliver procedures for the agreed national tariff set annually. Often, for procedures which required implants, devices, specialist consumables or equipment, private providers could not deliver services within tariff as they could not procure consumables at the same price as the NHS purchasing at volume, or could not sustain the volumes of patients needed to justify capital expenditure on specialist equipment.

Often choice was exercised by NHS providers on behalf of patients by agreeing to contract out set amounts of activity for certain procedures which were considered mutually cost-effective. Patients would then be contacted by the NHS Trust to ask if they would be prepared to have their procedure undertaken in the private hospital. This 'endorsement'

doubtless persuaded some patients who initially chose the more trusted and familiar brand of the NHS to have their procedure undertaken in the private sector. In parallel, and to protect and maximise income streams, NHS providers began to run significant volumes of additional theatre sessions using evenings and weekends to increase throughput, alongside a further increase in the use of day case surgery.
Little data exists on how many patients individually used choice, but Department of Health figures suggest that 200,000 elective procedures per annum were being undertaken in independent hospitals by 2011.[6] However, what is far less clear is how many of these procedures were undertaken as a result of individual patient choice, rather than contracting out by NHS providers. However, amongst other interventions, the use of choice whether instigated by individuals or by institutions, appeared to change provider behaviour and achieve notable waiting time reductions.

References

1. Department of Health. NHS Constitution. Department of Health; 2015. Available at: https://assets.publishing.service.gov.uk/government/uploads/system/uploads/attachment_data/file/480482/NHS_Constitution_WEB.pdf [accessed 23/09/2020)

2. Department of Health. Health and Social Care Act 2012. Department of Health; 2012. Available at: https://www.gov.uk/government/publications/health-and-social-care-act-2012-fact-sheets [accessed 23/09/2020]

3. Le Grand J. Motivation, Agency and Public Policy – of Knights and Knaves, Pawns and Queens. Oxford University Press: Oxford; 2013.

4. Le Grand J. The Other Invisible Hand – Delivering Public Services through Choice and Competition. Princeton University Press: Princeton and Oxford; 2007.

5. Robertson R, Dixon A. Choice at the point of referral: early results of a patient survey. The Kings Fund; 2009. Available at: https://www.kingsfund.org.uk/sites/default/files/Choice-at-the-point-of-referral-Kings-Fund-November-2009.pdf [accessed 23/09/2020]

6. Department of Health. Choice and Competition: Health and Social Care Act 2012, Department of Health; 2012. Available at: https://assets.publishing.service.gov.uk/government/uploads/system/uploads/attachment_data/file/138269/C4.-Factsheet-Choice-and-competition-270412.pdf [accessed 23/09/2020]
