# Supplementary material for: Outcomes for surgical procedures funded by the English health service but carried out in public versus independent hospitals: a database study
Source: BMJ Qual Saf. 2021 Sep 7;31(7):515–25. doi: 10.1136/bmjqs-2021-013522 (PMC9234423; doi:10.1136/bmjqs-2021-013522)
Supplement: Supplementary data [file bmjqs-2021-013522supp002.pdf]

Supplementary Table 1: Summary of studies comparing outcomes of elective surgery in English NHS hospitals and Independent Sector Hospital Providers

Ref	Study type	Data years	Procedures	Outcomes	Hospitals	Adjustment	Results summary
**Browne et al (BMC Health Services Research, 2008)**	Prospective cohort study	2006-2007	Hip or knee replacement, inguinal hernia repair, varicose vein surgery, cataract extraction	Change in patient-reported health status and health related quality of life. Patient-reported post-operative complications	6 ISHPs, 20 NHS providers	Disease severity, duration of symptoms, age, sex, socioeconomic status, previous similar surgery, general health, comorbidity	Mixed: no difference in most outcomes, but for some operations patients treated in ISHPs reported greater improvement, while for hernia repair patients treated in NHS hospitals reported greater improvement
**Chard et al (BMJ, 2011)**	Prospective cohort study	2008-2009	Hip or knee replacement, inguinal hernia repair, varicose vein surgery	Symptoms and disability reported by patients (Oxford hip and knee scores on a 48 point scale; Aberdeen varicose vein questionnaire) and quality of life (EuroQol EQ-5D score).	25 ISHPs, 72 NHS providers invited (not all chose to participate)	Adjusted for case-mix based on age, sex, socio-economic circumstances and preoperative questionnaire which asked about severity of symptoms and general health	Some outcomes were better in ISHPs, particularly for orthopaedic surgery.
**Siciliani et al (Health Econ, 2013)**	Retrospective cohort study	2006-2007	Hip replacement	Length of stay	179 NHS providers, 14 ISHPs	Age, gender, number and type of diagnoses, deprivation, regional variation	Shorter length of stay associated with treatment at an ISHP

**Appleby (BMJ 2020)**	Retrospective cohort study	2018-2019	Hip replacement	EQ-5D score	122 NHS providers, 90 ISHPs	PROMs risk-adjustment model (based on HES data)	Average health gain was slightly better among patients treated by non-NHS (independent) providers

Articles were identified using the following search terms in Google Scholar:

NHS AROUND(10) (private OR "independent provider" OR "independent sector") surgery operation hospital provider outcome

The first 40 abstracts were read to identify those studies pertaining to outcomes following surgery in English hospitals, with independent and NHS providers compared. All studies referenced in the following systematic review were similarly examined:

Kruse, F. M., Stadhouders, N. W., Adang, E. M., Groenewoud, S., & Jeurissen, P. (2018). Do private hospitals outperform public hospitals regarding efficiency, accessibility, and quality of care in the European Union? A literature review. *The International journal of health planning and management*, *33*(2), e434–e453. https://doi.org/10.1002/hpm.2502
