# Supplementary material for: Outcomes for surgical procedures funded by the English health service but carried out in public versus independent hospitals: a database study
Source: BMJ Qual Saf. 2021 Sep 7;31(7):515–25. doi: 10.1136/bmjqs-2021-013522 (PMC9234423; doi:10.1136/bmjqs-2021-013522)
Supplement: Supplementary data [file bmjqs-2021-013522supp003.pdf]

Supplementary Table 2: List of operations occurring at least 4000 times in both ISHPs and NHS hospitals between 01/04/2001 and 31/12/2019

OPCS code and name
A521 - Therapeutic lumbar epidural injection
A522 - Therapeutic sacral epidural injection
A528 - Other specified therapeutic epidural injection
A573 - Radiofrequency controlled thermal destruction of spinal nerve root
A577 - Injection of therapeutic substance around spinal nerve root
A604 - Radiofrequency controlled thermal destruction of peripheral nerve
A611 - Excision of lesion of peripheral nerve
A651 - Carpal tunnel release
A671 - Cubital tunnel release
A735 - Injection of therapeutic substance around peripheral nerve
C121 - Excision of lesion of eyelid NEC
C712 - Phacoemulsification of lens
C733 - Capsulotomy of posterior lens capsule
C751 - Insertion of prosthetic replacement for lens NEC
C794 - Injection into vitreous body NEC
C873 - Tomography evaluation of retina
D021 - Excision of lesion of external ear
D151 - Myringotomy with insertion of ventilation tube through tympanic membrane
E036 - Septoplasty of nose NEC
E081 - Polypectomy of internal nose
E091 - Excision of lesion of external nose
E142 - Intranasal ethmoidectomy
F091 - Surgical removal of impacted wisdom tooth
F093 - Surgical removal of wisdom tooth NEC
F094 - Surgical removal of tooth NEC
F095 - Surgical removal of retained root of tooth
F104 - Extraction of multiple teeth NEC
F109 - Unspecified simple extraction of tooth
F341 - Bilateral dissection tonsillectomy
G451 - Fibreoptic endoscopic examination of upper gastrointestinal tract and biopsy of lesion of upper gastrointestinal tract
G458 - Other specified diagnostic fibreoptic endoscopic examination of upper gastrointestinal tract
G459 - Unspecified diagnostic fibreoptic endoscopic examination of upper gastrointestinal tract
H201 - Fibreoptic endoscopic snare resection of lesion of colon
H202 - Fibreoptic endoscopic cauterisation of lesion of colon
H206 - Fibreoptic endoscopic resection of lesion of colon NEC
H221 - Diagnostic fibreoptic endoscopic examination of colon and biopsy of lesion of colon
H229 - Unspecified diagnostic endoscopic examination of colon
H231 - Endoscopic snare resection of lesion of lower bowel using fibreoptic sigmoidoscope
H251 - Diagnostic endoscopic examination of lower bowel and biopsy of lesion of lower bowel using fibreoptic sigmoidoscope
H259 - Unspecified diagnostic endoscopic examination of lower bowel using fibreoptic sigmoidoscope
H482 - Excision of skin tag of anus
H511 - Haemorrhoidectomy
H523 - Injection of sclerosing substance into haemorrhoid
H524 - Rubber band ligation of haemorrhoid
H568 - Other specified other operations on anus
J183 - Total cholecystectomy NEC
L703 - Ligation of artery NEC
L841 - Combined operations on primary long saphenous vein
L882 - Radiofrequency ablation of varicose vein of leg
M451 - Diagnostic endoscopic examination of bladder and biopsy of lesion of bladder NEC
M459 - Unspecified diagnostic endoscopic examination of bladder
M473 - Removal of urethral catheter from bladder
M494 - Introduction of therapeutic substance into bladder
M653 - Endoscopic resection of prostate NEC
M764 - Endoscopic dilation of urethra
M792 - Dilation of urethra NEC

N153 - Excision of lesion of epididymis
N171 - Bilateral vasectomy
N303 - Circumcision
O291 - Subacromial decompression
P054 - Excision of lesion of vulva NEC
P231 - Anterior and posterior colporrhaphy NEC
P232 - Anterior colporrhaphy NEC
P233 - Posterior colporrhaphy NEC
Q023 - Cauterisation of lesion of cervix uteri
Q074 - Total abdominal hysterectomy NEC
Q075 - Subtotal abdominal hysterectomy
Q089 - Unspecified vaginal excision of uterus
Q111 - Vacuum aspiration of products of conception from uterus NEC
Q121 - Introduction of intrauterine contraceptive device
Q165 - Radiofrequency ablation of endometrium
Q171 - Endoscopic resection of lesion of uterus
Q181 - Diagnostic endoscopic examination of uterus and biopsy of lesion of uterus
Q188 - Other specified diagnostic endoscopic examination of uterus
Q189 - Unspecified diagnostic endoscopic examination of uterus
Q491 - Endoscopic extirpation of lesion of ovary NEC
S065 - Excision of lesion of skin of head or neck NEC
S068 - Other specified other excision of lesion of skin
S069 - Unspecified other excision of lesion of skin
T202 - Primary repair of inguinal hernia using insert of prosthetic material
T209 - Unspecified primary repair of inguinal hernia
T212 - Repair of recurrent inguinal hernia using insert of prosthetic material
T242 - Repair of umbilical hernia using insert of prosthetic material
T243 - Repair of umbilical hernia using sutures
T252 - Primary repair of incisional hernia using insert of prosthetic material
T272 - Repair of ventral hernia using insert of prosthetic material
T273 - Repair of ventral hernia using sutures
T422 - Endoscopic destruction of lesion of peritoneum
T423 - Endoscopic division of adhesions of peritoneum
T439 - Unspecified diagnostic endoscopic examination of peritoneum
T521 - Palmar fasciectomy
T525 - Digital fasciectomy
T578 - Other specified other operations on fascia
T591 - Excision of ganglion of wrist
T592 - Excision of ganglion of hand NEC
T594 - Excision of ganglion of foot
T625 - Injection into bursa
T691 - Primary tenolysis
T723 - Release of constriction of sheath of tendon
T744 - Injection of therapeutic substance into tendon NEC
T791 - Plastic repair of rotator cuff of shoulder NEC
T962 - Excision of lesion of soft tissue NEC
V254 - Primary posterior laminectomy decompression of lumbar spine
V255 - Primary posterior decompression of lumbar spine NEC
V337 - Primary microdiscectomy of lumbar intervertebral disc
V485 - Radiofrequency controlled thermal denervation of spinal facet joint of lumbar vertebra
V544 - Injection around spinal facet of spine
W068 - Other specified total excision of bone
W082 - Excision of overgrowth of bone
W083 - Excision of excrescence of bone
W085 - Partial excision of bone NEC
W132 - Displacement osteotomy
W153 - Osteotomy of first metatarsal bone NEC
W157 - Osteotomy of bone of foot and fixation HFQ
W283 - Removal of internal fixation from bone NEC
W371 - Primary total prosthetic replacement of hip joint using cement
W381 - Primary total prosthetic replacement of hip joint not using cement
W391 - Primary total prosthetic replacement of hip joint NEC
W401 - Primary total prosthetic replacement of knee joint using cement

W411 - Primary total prosthetic replacement of knee joint not using cement
W421 - Primary total prosthetic replacement of knee joint NEC
W572 - Primary excision arthroplasty of joint NEC
W581 - Primary resurfacing arthroplasty of joint
W593 - Fusion of first metatarsophalangeal joint NEC
W595 - Fusion of interphalangeal joint of toe NEC
W621 - Primary arthrodesis and internal fixation of joint NEC
W712 - Open excision of intra-articular osteophyte
W742 - Reconstruction of intra-articular ligament NEC
W781 - Release of contracture of shoulder joint
W791 - Soft tissue correction of hallux valgus
W802 - Open debridement of joint NEC
W822 - Endoscopic resection of semilunar cartilage NEC
W833 - Endoscopic shaving of articular cartilage
W836 - Endoscopic excision of articular cartilage NEC
W851 - Endoscopic removal of loose body from knee joint
W858 - Other specified therapeutic endoscopic operations on cavity of knee joint
W879 - Unspecified diagnostic endoscopic examination of knee joint
W891 - Endoscopic chondroplasty NEC
W901 - Aspiration of joint
W902 - Arthrography
W903 - Injection of therapeutic substance into joint
W904 - Injection into joint NEC
W913 - Manipulation of prosthetic joint NEC
W919 - Unspecified other manipulation of joint
W931 - Primary hybrid prosthetic replacement of hip joint using cemented acetabular component
W941 - Primary hybrid prosthetic replacement of hip joint using cemented femoral component
X292 - Continuous intravenous infusion of therapeutic substance NEC
X362 - Venesection
X375 - Intramuscular injection for local action
X921 - Cytokine inhibitor drugs Band 1
