# Supplementary material for: Outcomes for surgical procedures funded by the English health service but carried out in public versus independent hospitals: a database study
Source: BMJ Qual Saf. 2021 Sep 7;31(7):515–25. doi: 10.1136/bmjqs-2021-013522 (PMC9234423; doi:10.1136/bmjqs-2021-013522)

SUPPLEMENTARY FIGURES

Supplementary Figure 1: Flow chart showing the data extraction and cleaning process for the selected operation types.

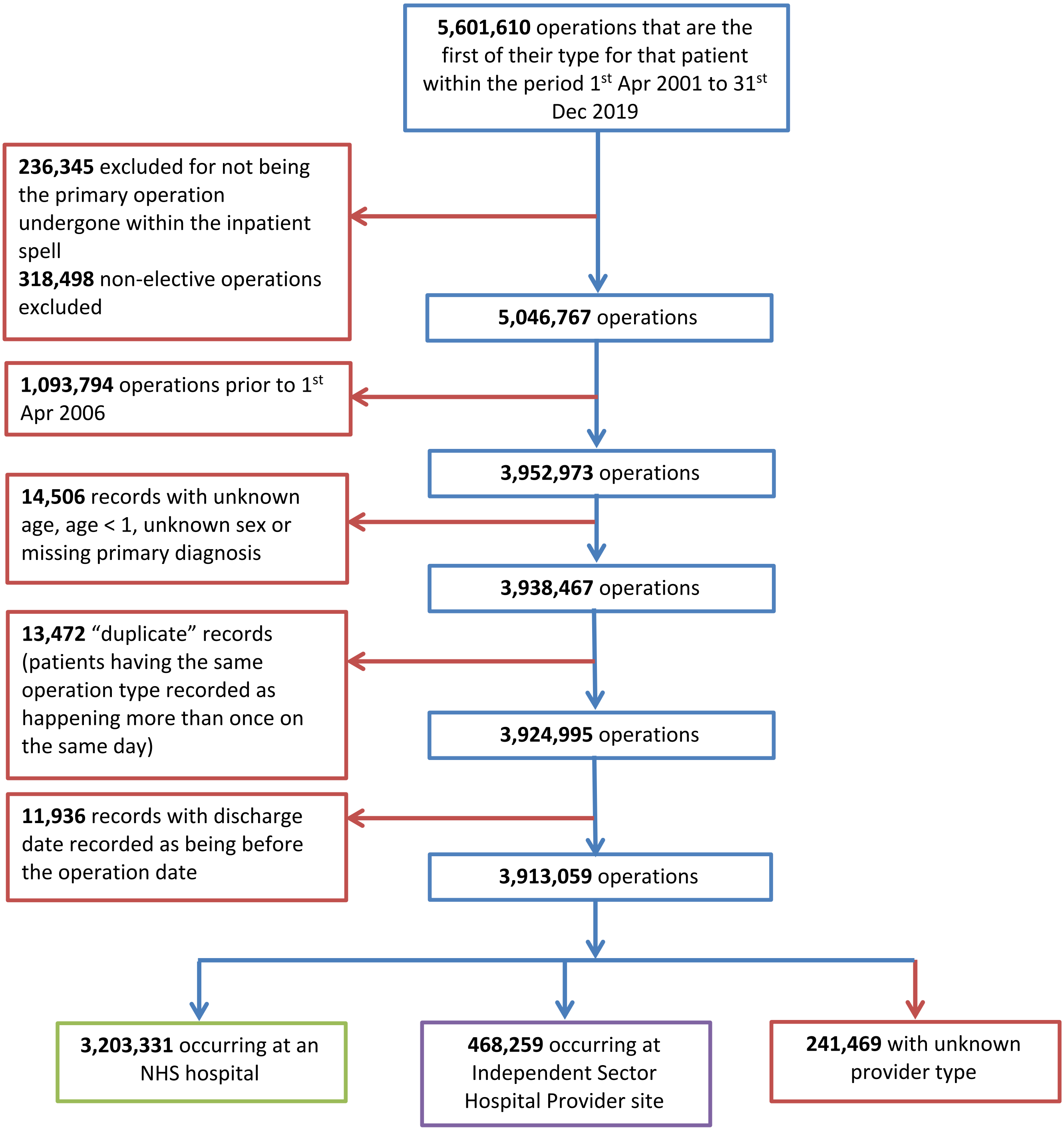

Supplement: Supplementary data [file bmjqs-2021-013522supp004.pdf]
