# Supplementary material for: Outcomes for surgical procedures funded by the English health service but carried out in public versus independent hospitals: a database study
Source: BMJ Qual Saf. 2021 Sep 7;31(7):515–25. doi: 10.1136/bmjqs-2021-013522 (PMC9234423; doi:10.1136/bmjqs-2021-013522)
Supplement: Supplementary data [file bmjqs-2021-013522supp005.pdf]

Supplementary Table 3: Operation specialties for selected operations

OPCS4 code	Description	Specialty*
**F091**	Surgical removal of impacted wisdom tooth	Oral Surgery, Maxillo-Facial Surgery
**F093**	Surgical removal of wisdom tooth NEC	Oral Surgery, Maxillo-Facial Surgery
**J183**	Total cholecystectomy NEC	Hepatobiliary & Pancreatic Surgery, Upper Gastrointestinal Surgery, General Surgery
**M653**	Endoscopic resection of prostate NEC	Urology
**Q074**	Total abdominal hysterectomy NEC	Gynaecology
**T212**	Repair of recurrent inguinal hernia using insert of prosthetic material	General Surgery
**T242**	Repair of umbilical hernia using insert of prosthetic material	General Surgery
**T243**	Repair of umbilical hernia using sutures	General Surgery
**T272**	Repair of ventral hernia using insert of prosthetic material	General Surgery
**V255**	Primary posterior decompression of lumbar spinal cord NEC	Spinal Surgery Service, Trauma & Orthopaedics, Neurosurgery
**W371**	Primary total prosthetic replacement of hip joint using cement	General Surgery, Trauma & Orthopaedics
**W381**	Primary total prosthetic replacement of hip joint not using cement	Trauma & Orthopaedics
**W391**	Primary total prosthetic replacement of hip joint NEC	Trauma & Orthopaedics
**W401**	Primary total prosthetic replacement of knee joint using cement	Trauma & Orthopaedics
**W411**	Primary total prosthetic replacement of knee joint not using cement	Trauma & Orthopaedics
**W421**	Primary total prosthetic replacement of knee joint NEC	Trauma & Orthopaedics
**W931**	Primary hybrid prosthetic replacement of hip joint using cemented acetabular component	Trauma & Orthopaedics
**W941**	Primary hybrid prosthetic replacement of hip joint using cemented femoral component	Trauma & Orthopaedics

*List of specialties comprises all those with > 1000 admissions and >1% of admissions when considering index admissions for this operation type. The specialty used was that within which the consultant was working during the period of care.
