# Supplementary material for: Outcomes for surgical procedures funded by the English health service but carried out in public versus independent hospitals: a database study
Source: BMJ Qual Saf. 2021 Sep 7;31(7):515–25. doi: 10.1136/bmjqs-2021-013522 (PMC9234423; doi:10.1136/bmjqs-2021-013522)
Supplement: Supplementary data [file bmjqs-2021-013522supp006.pdf]

Supplementary Table 4: List of ISHPs conducting at least 10 of the selected operations during the study period. Note that if a hospital changed ownership during the study period it may appear more than once in the table. Note also that not all hospitals listed here were operating as ISHPs during the whole study period. ISHPs are classified as for profit or not-for-profit.

Organisation name and code	Number of ops in data set	Profit/non-profit	Owner
NXM01 - THE HORDER CENTRE - ST JOHNS ROAD	10,000 - 19,999	NON-PROFIT	Other
NTP11 - SOUTHAMPTON NHS TREATMENT CENTRE	10,000 - 19,999	PROFIT	Practice Plus
NVC20 - THE YORKSHIRE CLINIC	5,000 - 9,999	PROFIT	Ramsay
NVC06 - FITZWILLIAM HOSPITAL	5,000 - 9,999	PROFIT	Ramsay
NTP13 - BARLBOROUGH NHS TREATMENT CENTRE	5,000 - 9,999	PROFIT	Practice Plus
NTP15 - NORTH EAST LONDON TREATMENT CENTRE CARE UK	5,000 - 9,999	PROFIT	Practice Plus
NVC40 - WOODTHORPE HOSPITAL	5,000 - 9,999	PROFIT	Ramsay
NVC23 - WOODLAND HOSPITAL	5,000 - 9,999	PROFIT	Ramsay
NT351 - SPIRE HULL AND EAST RIDING HOSPITAL	5,000 - 9,999	PROFIT	Spire
NVC18 - SPRINGFIELD HOSPITAL	5,000 - 9,999	PROFIT	Ramsay
NVC04 - DUCHY HOSPITAL	5,000 - 9,999	PROFIT	Ramsay
NVC25 - HORTON NHS TREATMENT CENTRE	5,000 - 9,999	PROFIT	Ramsay
NVC07 - FULWOOD HALL HOSPITAL	5,000 - 9,999	PROFIT	Ramsay
NVC28 - CLIFTON PARK HOSPITAL	5,000 - 9,999	PROFIT	Ramsay
NT333 - SPIRE WASHINGTON HOSPITAL	5,000 - 9,999	PROFIT	Spire
NVC19 - RIVERS HOSPITAL	5,000 - 9,999	PROFIT	Ramsay
NT318 - SPIRE NORWICH HOSPITAL	5,000 - 9,999	PROFIT	Spire
NTPH2 - EMERSONS GREEN NHS TREATMENT CENTRE	5,000 - 9,999	PROFIT	Practice Plus
NT401 - BMI - THE ALEXANDRA HOSPITAL	5,000 - 9,999	PROFIT	BMI
NT225 - NUFFIELD HEALTH, LEEDS HOSPITAL	5,000 - 9,999	NON-PROFIT	Nuffield
NWF01 - BENENDEN HOSPITAL	2,000 - 4,999	NON-PROFIT	Other
NT226 - NUFFIELD HEALTH, LEICESTER HOSPITAL	2,000 - 4,999	NON-PROFIT	Nuffield
NTE02 - ST HUGH'S HOSPITAL	2,000 - 4,999	NON-PROFIT	Other
NT213 - NUFFIELD HEALTH, DERBY HOSPITAL	2,000 - 4,999	NON-PROFIT	Nuffield
NT237 - NUFFIELD HEALTH, TEES HOSPITAL	2,000 - 4,999	NON-PROFIT	Nuffield
NT233 - NUFFIELD HEALTH, PLYMOUTH HOSPITAL	2,000 - 4,999	NON-PROFIT	Nuffield
NT230 - NUFFIELD HEALTH, NORTH STAFFORDSHIRE HOSPITAL	2,000 - 4,999	NON-PROFIT	Nuffield
NV313 - CIRCLE - NOTTINGHAM NHS TREATMENT CENTRE	2,000 - 4,999	PROFIT	Circle
NVC13 - OAKS HOSPITAL	2,000 - 4,999	PROFIT	Ramsay
NVC05 - EUXTON HALL HOSPITAL	2,000 - 4,999	PROFIT	Ramsay
NVC08 - MOUNT STUART HOSPITAL	2,000 - 4,999	PROFIT	Ramsay
NT350 - SPIRE METHLEY PARK HOSPITAL	2,000 - 4,999	PROFIT	Spire
NVC12 - OAKLANDS HOSPITAL	2,000 - 4,999	PROFIT	Ramsay
NT420 - BMI - THE HIGHFIELD HOSPITAL	2,000 - 4,999	PROFIT	BMI
NT457 - BMI WOODLANDS HOSPITAL	2,000 - 4,999	PROFIT	BMI
NVC09 - NEW HALL HOSPITAL	2,000 - 4,999	PROFIT	Ramsay
NVC21 - WEST MIDLANDS HOSPITAL	2,000 - 4,999	PROFIT	Ramsay
NV302 - CIRCLE BATH HOSPITAL	2,000 - 4,999	PROFIT	Circle
NT348 - SPIRE ELLAND HOSPITAL	2,000 - 4,999	PROFIT	Spire
NT337 - SPIRE LIVERPOOL HOSPITAL	2,000 - 4,999	PROFIT	Spire
NT417 - BMI - GORING HALL HOSPITAL	2,000 - 4,999	PROFIT	BMI

NVC22 - WINFIELD HOSPITAL	2,000 - 4,999	PROFIT	Ramsay
NVC15 - PINEHILL HOSPITAL	2,000 - 4,999	PROFIT	Ramsay
NT441 - BMI - THREE SHIRES HOSPITAL	2,000 - 4,999	PROFIT	BMI
NT332 - SPIRE LEEDS HOSPITAL	2,000 - 4,999	PROFIT	Spire
NT321 - SPIRE LITTLE ASTON HOSPITAL	2,000 - 4,999	PROFIT	Spire
NVC16 - RENACRES HOSPITAL	2,000 - 4,999	PROFIT	Ramsay
NT347 - SPIRE FYLDE COAST HOSPITAL	2,000 - 4,999	PROFIT	Spire
NT313 - SPIRE WELLESLEY HOSPITAL	2,000 - 4,999	PROFIT	Spire
NT322 - SPIRE LEICESTER HOSPITAL	2,000 - 4,999	PROFIT	Spire
NT324 - SPIRE CHESHIRE HOSPITAL	2,000 - 4,999	PROFIT	Spire
NT320 - SPIRE PARKWAY HOSPITAL	2,000 - 4,999	PROFIT	Spire
NVC17 - ROWLEY HALL HOSPITAL	2,000 - 4,999	PROFIT	Ramsay
NT305 - SPIRE PORTSMOUTH HOSPITAL	2,000 - 4,999	PROFIT	Spire
NT424 - BMI - THE MERIDEN HOSPITAL	2,000 - 4,999	PROFIT	BMI
NT304 - SPIRE SOUTHAMPTON HOSPITAL	2,000 - 4,999	PROFIT	Spire
NT301 - SPIRE SOUTH BANK HOSPITAL	2,000 - 4,999	PROFIT	Spire
NTC01 - SHEPTON MALLET NHS TREATMENT CENTRE	2,000 - 4,999	PROFIT	UK Specialist hospitals
NT402 - BMI - BATH CLINIC	2,000 - 4,999	PROFIT	BMI
NT412 - BMI - THE DROITWICH SPA HOSPITAL	2,000 - 4,999	PROFIT	BMI
NT448 - BMI THE HUDDERSFIELD HOSPITAL	2,000 - 4,999	PROFIT	BMI
NT430 - BMI - THE RIDGEWAY HOSPITAL	2,000 - 4,999	PROFIT	BMI
NTPH1 - SHEPTON MALLET NHS TREATMENT CENTRE	2,000 - 4,999	PROFIT	Practice Plus
NT312 - SPIRE ALEXANDRA HOSPITAL	2,000 - 4,999	PROFIT	Spire
NTD02 - THE CHESHIRE & MERSEYSIDE NHS TREATMENT CENTRE	2,000 - 4,999	PROFIT	Interhealth care services
NT422 - BMI - THE LONDON INDEPENDENT HOSPITAL	2,000 - 4,999	PROFIT	BMI
NT432 - BMI - THE SANDRINGHAM HOSPITAL	2,000 - 4,999	PROFIT	BMI
NVC11 - NORTH DOWNS HOSPITAL	2,000 - 4,999	PROFIT	Ramsay
NV323 - CIRCLE READING HOSPITAL	2,000 - 4,999	PROFIT	Circle
NVG01 - FAIRFIELD HOSPITAL	2,000 - 4,999	PROFIT	Ramsay
NT325 - SPIRE MURRAYFIELD HOSPITAL	2,000 - 4,999	PROFIT	Spire
NT427 - BMI - THE PARK HOSPITAL	2,000 - 4,999	PROFIT	BMI
NT302 - SPIRE BRISTOL HOSPITAL	2,000 - 4,999	PROFIT	Spire
NTP17 - SUSSEX ORTHOPAEDIC NHS TREATMENT CENTRE	2,000 - 4,999	PROFIT	Practice Plus
NT410 - BMI - THE CHILTERN HOSPITAL	2,000 - 4,999	PROFIT	BMI
NT450 - BMI THE LINCOLN HOSPITAL	2,000 - 4,999	PROFIT	BMI
NVC02 - THE BERKSHIRE INDEPENDENT HOSPITAL	2,000 - 4,999	PROFIT	Ramsay
NT308 - SPIRE GATWICK PARK HOSPITAL	2,000 - 4,999	PROFIT	Spire
NT316 - SPIRE HARPENDEN HOSPITAL	2,000 - 4,999	PROFIT	Spire
NT411 - BMI - THE CLEMENTINE CHURCHILL HOSPITAL	2,000 - 4,999	PROFIT	BMI
NT418 - BMI - THE HAMPSHIRE CLINIC	2,000 - 4,999	PROFIT	BMI
NT314 - SPIRE LONDON EAST	2,000 - 4,999	PROFIT	Spire
NT364 - SPIRE MONTEFIORE HOSPITAL	2,000 - 4,999	PROFIT	Spire
NT403 - BMI - THE BEARDWOOD HOSPITAL	2,000 - 4,999	PROFIT	BMI
NT446 - BMI ST EDMUNDS HOSPITAL	2,000 - 4,999	PROFIT	BMI
NT222 - NUFFIELD HEALTH, IPSWICH HOSPITAL	1,000 - 1,999	NON-PROFIT	Nuffield
NT209 - NUFFIELD HEALTH, CAMBRIDGE HOSPITAL	1,000 - 1,999	NON-PROFIT	Nuffield
NT212 - NUFFIELD HEALTH, CHICHESTER HOSPITAL	1,000 - 1,999	NON-PROFIT	Nuffield
NTE03 - CLAREMONT HOSPITAL	1,000 - 1,999	NON-PROFIT	Other

NT210 - NUFFIELD HEALTH, THE GROSVENOR HOSPITAL, CHESTER	1,000 - 1,999	NON-PROFIT	Nuffield
NT229 - NUFFIELD HEALTH, NEWCASTLE UPON TYNE HOSPITAL	1,000 - 1,999	NON-PROFIT	Nuffield
NT219 - NUFFIELD HEALTH, HEREFORD HOSPITAL	1,000 - 1,999	NON-PROFIT	Nuffield
NT215 - NUFFIELD HEALTH, EXETER HOSPITAL	1,000 - 1,999	NON-PROFIT	Nuffield
NT242 - NUFFIELD HEALTH, WOLVERHAMPTON HOSPITAL	1,000 - 1,999	NON-PROFIT	Nuffield
NT238 - NUFFIELD HEALTH, TAUNTON HOSPITAL	1,000 - 1,999	NON-PROFIT	Nuffield
NT204 - NUFFIELD HEALTH, BRENTWOOD HOSPITAL	1,000 - 1,999	NON-PROFIT	Nuffield
NT245 - NUFFIELD HEALTH, YORK HOSPITAL	1,000 - 1,999	NON-PROFIT	Nuffield
NT214 - NUFFIELD HEALTH, WESSEX HOSPITAL	1,000 - 1,999	NON-PROFIT	Nuffield
NT404 - BMI - THE BEAUMONT HOSPITAL	1,000 - 1,999	PROFIT	BMI
NT339 - SPIRE REGENCY HOSPITAL	1,000 - 1,999	PROFIT	Spire
NVC44 - THE WESTBOURNE CENTRE	1,000 - 1,999	PROFIT	Ramsay
NVC01 - ASHTEAD HOSPITAL	1,000 - 1,999	PROFIT	Ramsay
NT443 - BMI - THE WINTERBOURNE HOSPITAL	1,000 - 1,999	PROFIT	BMI
NYW01 - ASPEN - THE HOLLY	1,000 - 1,999	PROFIT	Aspen healthcare
NVC35 - TEES VALLEY TREATMENT CENTRE	1,000 - 1,999	PROFIT	Ramsay
NT327 - SPIRE MANCHESTER HOSPITAL	1,000 - 1,999	PROFIT	Spire
ADP02 - KIMS HOSPITAL (NEWNHAM COURT)	1,000 - 1,999	PROFIT	KIMS
NTPH3 - DEVIZES NHS TREATMENT CENTRE	1,000 - 1,999	PROFIT	Practice Plus
NT317 - SPIRE CAMBRIDGE LEA HOSPITAL	1,000 - 1,999	PROFIT	Spire
NT439 - BMI - THE SOUTH CHESHIRE PRIVATE HOSPITAL	1,000 - 1,999	PROFIT	BMI
NT309 - SPIRE SUSSEX HOSPITAL	1,000 - 1,999	PROFIT	Spire
NT346 - SPIRE ST SAVIOURS HOSPITAL	1,000 - 1,999	PROFIT	Spire
NT451 - BMI THE CAVELL HOSPITAL	1,000 - 1,999	PROFIT	BMI
NVC24 - BODMIN NHS TREATMENT CENTRE	1,000 - 1,999	PROFIT	Ramsay
NVC14 - PARK HILL HOSPITAL	1,000 - 1,999	PROFIT	Ramsay
NTA04 - NOTTINGHAM NHS TREATMENT CENTRE (NATIONS HEALTHCARE)	1,000 - 1,999	PROFIT	Nations healthcare
NT604 - SUSSEX ORTHOPAEDIC NHS TREATMENT CENTRE	1,000 - 1,999	PROFIT	Care UK
NT319 - SPIRE HARTSWOOD HOSPITAL	1,000 - 1,999	PROFIT	Spire
NT445 - BMI THE EDGBASTON HOSPITAL	1,000 - 1,999	PROFIT	BMI
NT440 - BMI - THORNBURY HOSPITAL	1,000 - 1,999	PROFIT	BMI
NTX01 - ONE HEALTH GROUP LTD	1,000 - 1,999	PROFIT	One Health
NTPH5 - PENINSULA NHS TREATMENT CENTRE	1,000 - 1,999	PROFIT	Practice Plus
NT344 - SPIRE DUNEDIN HOSPITAL	1,000 - 1,999	PROFIT	Spire
NVC34 - FYLDE COAST NHS TREATMENT CENTRE	1,000 - 1,999	PROFIT	Ramsay
NT434 - BMI - THE SAXON CLINIC	1,000 - 1,999	PROFIT	BMI
NT315 - SPIRE BUSHEY HOSPITAL	1,000 - 1,999	PROFIT	Spire
NT497 - BMI GISBURNE PARK HOSPITAL	1,000 - 1,999	PROFIT	BMI
NT436 - BMI - SHIRLEY OAKS HOSPITAL	1,000 - 1,999	PROFIT	BMI
NVC33 - GISBURNE PARK NHS TREATMENT CENTRE	1,000 - 1,999	PROFIT	Ramsay
NT449 - BMI THE LANCASTER HOSPITAL	1,000 - 1,999	PROFIT	BMI
NT408 - BMI - THE CHAUCER HOSPITAL	1,000 - 1,999	PROFIT	BMI
NYW04 - ASPEN - CLAREMONT HOSPITAL	1,000 - 1,999	PROFIT	Aspen healthcare
NT447 - BMI THE DUCHY HOSPITAL	1,000 - 1,999	PROFIT	BMI
NTX11 - ONE HEALTH GROUP CLINIC - THORNBURY	1,000 - 1,999	PROFIT	One Health
NT413 - BMI - THE ESPERANCE HOSPITAL	1,000 - 1,999	PROFIT	BMI

NT714 - GREATER MANCHESTER SURGICAL CENTRE	1,000 - 1,999	PROFIT	Netcare
NT438 - BMI - THE SOMERFIELD HOSPITAL	1,000 - 1,999	PROFIT	BMI
NT345 - SPIRE CLARE PARK HOSPITAL	1,000 - 1,999	PROFIT	Spire
NT433 - BMI - SARUM ROAD HOSPITAL	1,000 - 1,999	PROFIT	BMI
NT206 - NUFFIELD HEALTH, BRISTOL HOSPITAL (CHESTERFIELD)	500 - 999	NON-PROFIT	Nuffield
NT235 - NUFFIELD HEALTH, SHREWSBURY HOSPITAL	500 - 999	NON-PROFIT	Nuffield
NT224 - NUFFIELD HEALTH, WARWICKSHIRE HOSPITAL	500 - 999	NON-PROFIT	Nuffield
NT218 - NUFFIELD HEALTH, HAYWARDS HEATH HOSPITAL	500 - 999	NON-PROFIT	Nuffield
NT244 - NUFFIELD HOSPITAL OXFORD (THE MANOR)	500 - 999	NON-PROFIT	Nuffield
NVC0R - TEES VALLEY HOSPITAL	500 - 999	PROFIT	Ramsay
NT406 - BMI - THE BLACKHEATH HOSPITAL	500 - 999	PROFIT	BMI
NT419 - BMI - THE HARBOUR HOSPITAL	500 - 999	PROFIT	BMI
NT421 - BMI - THE KINGS OAK HOSPITAL	500 - 999	PROFIT	BMI
NT310 - SPIRE TUNBRIDGE WELLS HOSPITAL	500 - 999	PROFIT	Spire
NT435 - BMI - THE SHELBURNE HOSPITAL	500 - 999	PROFIT	BMI
NT423 - BMI - THE MANOR HOSPITAL	500 - 999	PROFIT	BMI
NT405 - BMI - BISHOPS WOOD	500 - 999	PROFIT	BMI
NT414 - BMI - FAWKHAM MANOR HOSPITAL	500 - 999	PROFIT	BMI
NT409 - BMI - CHELSFIELD PARK HOSPITAL	500 - 999	PROFIT	BMI
NT429 - BMI - THE PRIORY HOSPITAL	500 - 999	PROFIT	BMI
NTA03 - THE MIDLANDS NHS TREATMENT CENTRE	500 - 999	PROFIT	Nations healthcare
NTYF1 - SPENCER PRIVATE HOSPITALS	500 - 999	PROFIT	Other
NT431 - BMI - THE RUNNYMEDE HOSPITAL	500 - 999	PROFIT	BMI
NVC39 - KENDAL NHS TREATMENT CENTRE	500 - 999	PROFIT	Ramsay
NT331 - BUPA HILL AND EAST RIDING HOSPITAL	500 - 999	PROFIT	Spire
NW931 - CLINICENTA SURGICENTRE	500 - 999	PROFIT	Clinicenta
NT428 - BMI - THE PRINCESS MARGARET HOSPITAL	500 - 999	PROFIT	BMI
NTX12 - ONE HEALTH GROUP CLINIC - CLAREMONT	500 - 999	PROFIT	One Health
NTPAD - ST MARY'S NHS TREATMENT CENTRE	500 - 999	PROFIT	Practice Plus
NT211 - NUFFIELD HEALTH, CHELTENHAM HOSPITAL	200 - 499	NON-PROFIT	Nuffield
NXM04 - THE MCINDOE CENTRE	200 - 499	NON-PROFIT	Other
8F926 - THE NOTTINGHAM NUFFIELD HOSPITAL	200 - 499	NON-PROFIT	Nuffield
AAH01 - TETBURY HOSPITAL TRUST	200 - 499	NON-PROFIT	Other
NT241 - NUFFIELD HEALTH, WOKING HOSPITAL	200 - 499	NON-PROFIT	Nuffield
NT205 - NUFFIELD HEALTH, BRIGHTON HOSPITAL	200 - 499	NON-PROFIT	Nuffield
NT202 - NUFFIELD HEALTH, BOURNEMOUTH HOSPITAL	200 - 499	NON-PROFIT	Nuffield
NT239 - NUFFIELD HEALTH, TUNBRIDGE WELLS HOSPITAL	200 - 499	NON-PROFIT	Nuffield
NNQ01 - BRAINTREE COMMUNITY HOSPITAL	200 - 499	PROFIT	Spencer
NT343 - SPIRE THAMES VALLEY HOSPITAL	200 - 499	PROFIT	Spire
NTP16 - WILL ADAMS NHS TREATMENT CENTRE	200 - 499	PROFIT	Practice Plus
NT506 - NORTH EAST LONDON NHS TREATMENT CENTRE	200 - 499	PROFIT	Care UK
NVC10 - NEW HALL NHS TREATMENT CENTRE	200 - 499	PROFIT	Ramsay
NVC29 - COBALT HOSPITAL	200 - 499	PROFIT	Ramsay
NT437 - BMI - THE SLOANE HOSPITAL	200 - 499	PROFIT	BMI

NTP23 - ECCLESHILL NHS TREATMENT CENTRE	200 - 499	PROFIT	Practice Plus
NT349 - SPIRE LONGLANDS CONSULTING ROOMS	200 - 499	PROFIT	Spire
NYW02 - ASPEN - PARKSIDE HOSPITAL	200 - 499	PROFIT	Aspen healthcare
NT455 - BMI MOUNT ALVERNIA HOSPITAL	200 - 499	PROFIT	BMI
NAM01 - PROBUS SURGICAL CENTRE	200 - 499	PROFIT	Probus surgery
NT30A - SPIRE NOTTINGHAM HOSPITAL	200 - 499	PROFIT	Spire
NVC31 - BLAKELANDS HOSPITAL	200 - 499	PROFIT	Ramsay
NT416 - BMI - HENDON HOSPITAL	200 - 499	PROFIT	BMI
NYW03 - ASPEN - HIGHGATE HOSPITAL	200 - 499	PROFIT	Aspen healthcare
NT3X3 - SPIRE ST ANTHONY'S HOSPITAL	200 - 499	PROFIT	Spire
NTYE7 - THE HORDER CENTRE	100 - 199	NON-PROFIT	Other
NXP04 - HATHAWAY MEDICAL CENTRE	100 - 199	PROFIT	Independent health group
NTA01 - ECCLESHILL NHS TREATMENT CENTRE	100 - 199	PROFIT	Nations healthcare
8DD07 - BUPA NORTH CHESHIRE HOSPITAL	100 - 199	PROFIT	Bupa
NT415 - BMI - THE FOSCOTE HOSPITAL	100 - 199	PROFIT	BMI
NT338 - SPIRE YALE HOSPITAL	100 - 199	PROFIT	Spire
NVC27 - BOSTON WEST HOSPITAL	100 - 199	PROFIT	Ramsay
NT602 - WILL ADAMS NHS TREATMENT CENTRE	100 - 199	PROFIT	Care UK
NTP03 - MILNROW VILLAGE PRACTICE (ICATS)	100 - 199	PROFIT	Practice Plus
NTH09 - HULL AND EAST RIDING HOSPITAL	100 - 199	PROFIT	Classic hospitals
8A643 - THE RIVERS HOSPITAL	100 - 199	PROFIT	Other
NXP17 - WHITE HORSE HEALTH CENTRE - IHG	100 - 199	PROFIT	Independent health group
NTY48 - TETBURY HOSPITAL	10 - 99	NON-PROFIT	Other
NT207 - NUFFIELD HEALTH, BRISTOL HOSPITAL (ST MARY'S)	10 - 99	NON-PROFIT	Nuffield
NTY57 - BENENDEN HOSPITAL	10 - 99	NON-PROFIT	Other
NT221 - NUFFIELD HEALTH, HULL HOSPITAL	10 - 99	NON-PROFIT	Nuffield
NTP14 - MID KENT NHS TREATMENT CENTRE	10 - 99	PROFIT	Practice Plus
8A805 - HOLLY HOUSE HOSPITAL	10 - 99	PROFIT	Other
8AY29 - BMI THE PARK HOSPITAL	10 - 99	PROFIT	BMI
NTX51 - ONE HEALTH GROUP - BARLBOROUGH TREATMENT CENTRE	10 - 99	PROFIT	One Health
NT340 - BMI SOUTH CHESHIRE PRIVATE HOSPITAL (SPIRE)	10 - 99	PROFIT	Spire
NTYD5 - WOODLANDS HOSPITAL	10 - 99	PROFIT	Other
NT603 - ST MARY'S NHS TREATMENT CENTRE	10 - 99	PROFIT	Care UK
NTX19 - HUDDERSFIELD HOSPITAL - THE ONE HEALTH GROUP	10 - 99	PROFIT	One Health
NT808 - CAPIO NEW HALL HOSPITAL NHS TREATMENT CENTRE	10 - 99	PROFIT	Ramsay
NT503 - MID KENT NHS TREATMENT CENTRE	10 - 99	PROFIT	Care UK
NED30 - ASHTEAD HOSPITAL	10 - 99	PROFIT	EDICS
NNE02 - DORKING GENERAL HOSPITAL	10 - 99	PROFIT	Spencer
NXP02 - OLD TOWN SURGERY	10 - 99	PROFIT	Independent health group
NTH03 - ST SAVIOUR'S HOSPITAL	10 - 99	PROFIT	Classic hospitals
NT425 - BMI - THE NUNEATON PRIVATE HOSPITAL	10 - 99	PROFIT	BMI

NTH06 - ELLAND HOSPITAL	10 - 99	PROFIT	Classic hospitals
NXP43 - MILLSTREAM HOUSE	10 - 99	PROFIT	Independent health group
NT456 - BMI THE CASTLE CONSULTING CENTRE	10 - 99	PROFIT	BMI
NT809 - CAPIO NORTH DOWNS HOSPITAL	10 - 99	PROFIT	Ramsay
8A917 - BUPA RODING HOSPITAL	10 - 99	PROFIT	Bupa
NTH11 - LOURDES HOSPITAL	10 - 99	PROFIT	Classic hospitals
8A517 - BMI MANOR HOSPITAL	10 - 99	PROFIT	BMI
NT490 - BMI SOUTHEND PRIVATE HOSPITAL	10 - 99	PROFIT	BMI
NTH08 - METHLEY PARK HOSPITAL	10 - 99	PROFIT	Classic hospitals
NTY25 - HOLLY HOUSE HOSPITAL	10 - 99	PROFIT	Other
NEY01 - PIONEER HEALTHCARE LTD - CLAREMONT HOSPITAL	10 - 99	PROFIT	Pioneer
NEY14 - OAKLANDS HOSPITAL	10 - 99	PROFIT	Pioneer
NT407 - BMI - CHATSWORTH SUITE	10 - 99	PROFIT	BMI
