# Supplementary material for: Outcomes for surgical procedures funded by the English health service but carried out in public versus independent hospitals: a database study
Source: BMJ Qual Saf. 2021 Sep 7;31(7):515–25. doi: 10.1136/bmjqs-2021-013522 (PMC9234423; doi:10.1136/bmjqs-2021-013522)
Supplement: Supplementary data [file bmjqs-2021-013522supp007.pdf]

Supplementary Table 5: Coefficients from propensity score models (logistic regression models)

F091	Wisdom tooth impacted (org type=="ISHP")		
Predictors	*Odds Ratios*	*CI*	*p*
(Intercept)	0.00	0.00 – 0.00	<0.001
Sex [Female]	0.95	0.92 – 0.98	0.001
IMD Quintile [5]	(ref)		
IMD Quintile [4]	0.82	0.78 – 0.86	<0.001
IMD Quintile [3]	0.84	0.80 – 0.88	<0.001
IMD Quintile [2]	0.77	0.74 – 0.81	<0.001
IMD Quintile [1]	0.83	0.79 – 0.88	<0.001
IMD Quintile [Unknown]	0.70	0.57 – 0.86	0.001
Age	1.01	1.01 – 1.01	<0.001
Comorbidity score	0.99	0.98 – 1.00	0.147
Ethnicity [White]	(ref)		
Ethnicity [Asian]	0.84	0.78 – 0.90	<0.001
Ethnicity [Black]	0.40	0.36 – 0.46	<0.001
Ethnicity [Mixed]	0.89	0.77 – 1.02	0.106
Ethnicity [Other/Unknown]	1.15	1.11 – 1.19	<0.001
Year [2006]	(ref)		
Year [2007]	0.53	0.35 – 0.80	0.002
Year [2008]	1.71	1.25 – 2.39	0.001
Year [2009]	6.00	4.51 – 8.17	<0.001
Year [2010]	11.92	9.02 – 16.14	<0.001
Year [2011]	20.71	15.72 – 27.98	<0.001
Year [2012]	17.94	13.61 – 24.26	<0.001
Year [2013]	27.57	20.95 – 37.24	<0.001
Year [2014]	15.76	11.95 – 21.32	<0.001
Year [2015]	19.14	14.52 – 25.88	<0.001
Year [2016]	12.38	9.36 – 16.78	<0.001
Year [2017]	23.03	17.49 – 31.13	<0.001
Year [2018]	33.15	25.20 – 44.77	<0.001
Year [2019]	36.81	27.98 – 49.71	<0.001

F093	Wisdom tooth NEC (org type=="ISHP")		
Predictors	*Odds Ratios*	*CI*	*p*
(Intercept)	0	0.00 – 0.00	<0.001
Sex [Female]	0.88	0.85 – 0.90	<0.001
IMD Quintile [5]	(ref)		
IMD Quintile [4]	0.84	0.80 – 0.89	<0.001
IMD Quintile [3]	0.85	0.81 – 0.89	<0.001
IMD Quintile [2]	0.82	0.78 – 0.86	<0.001
IMD Quintile [1]	0.73	0.69 – 0.77	<0.001
IMD Quintile [Unknown]	0.54	0.42 – 0.67	<0.001
Age	1.01	1.01 – 1.01	<0.001
Comorbidity score	0.95	0.94 – 0.96	<0.001
Ethnicity [White]	(ref)		
Ethnicity [Asian]	0.79	0.73 – 0.85	<0.001
Ethnicity [Black]	0.43	0.38 – 0.48	<0.001
Ethnicity [Mixed]	0.83	0.71 – 0.96	0.013
Ethnicity [Other/Unknown]	1.15	1.11 – 1.20	<0.001
Year [2006]	(ref)		
Year [2007]	0.76	0.51 – 1.13	0.168
Year [2008]	1.72	1.25 – 2.43	0.001
Year [2009]	2.06	1.51 – 2.88	<0.001
Year [2010]	6.57	4.93 – 8.98	<0.001
Year [2011]	15.49	11.70 – 21.08	<0.001
Year [2012]	17.84	13.48 – 24.26	<0.001
Year [2013]	15.39	11.62 – 20.94	<0.001
Year [2014]	23.49	17.76 – 31.91	<0.001
Year [2015]	14.01	10.58 – 19.06	<0.001
Year [2016]	6.7	5.03 – 9.15	<0.001
Year [2017]	14.71	11.10 – 20.01	<0.001
Year [2018]	19.89	15.03 – 27.05	<0.001
Year [2019]	24.87	18.79 – 33.81	<0.001

J183	Cholecystectomy (org type=="ISHP")		
Predictors	*Odds Ratios*	*CI*	*p*
(Intercept)	0.02	0.02 – 0.02	<0.001
Sex [Female]	1.39	1.34 – 1.45	<0.001
IMD Quintile [5]	(ref)		
IMD Quintile [4]	0.96	0.91 – 1.00	0.053
IMD Quintile [3]	0.85	0.82 – 0.89	<0.001
IMD Quintile [2]	0.73	0.70 – 0.76	<0.001
IMD Quintile [1]	0.62	0.59 – 0.65	<0.001
IMD Quintile [Unknown]	0.19	0.13 – 0.27	<0.001
Age	0.99	0.99 – 0.99	<0.001
Comorbidity score	0.95	0.94 – 0.95	<0.001
Ethnicity [White]	(ref)		
Ethnicity [Asian]	0.72	0.67 – 0.79	<0.001
Ethnicity [Black]	0.47	0.40 – 0.56	<0.001
Ethnicity [Mixed]	0.88	0.72 – 1.07	0.224
Ethnicity [Other/Unknown]	2.21	2.13 – 2.30	<0.001
Year [2006]	(ref)		
Year [2007]	0.73	0.59 – 0.91	0.004
Year [2008]	1.12	0.92 – 1.37	0.245
Year [2009]	2.62	2.20 – 3.14	<0.001
Year [2010]	3.45	2.92 – 4.11	<0.001
Year [2011]	4.25	3.60 – 5.05	<0.001
Year [2012]	5.65	4.81 – 6.70	<0.001
Year [2013]	6.05	5.15 – 7.17	<0.001
Year [2014]	6.96	5.93 – 8.23	<0.001
Year [2015]	7.31	6.22 – 8.64	<0.001
Year [2016]	7.45	6.34 – 8.81	<0.001
Year [2017]	8.18	6.97 – 9.67	<0.001
Year [2018]	8.95	7.63 – 10.58	<0.001
Year [2019]	8.7	7.41 – 10.29	<0.001

Supplementary Table 5: Coefficients from propensity score models (logistic regression models)

	**UH repair (prosthetics) (org type=="ISHP")**				**UH repair (sutures) (org type=="ISHP")**				**VH repair (prosthetics) (org type=="ISHP")**		
**T242**				**T243**				**T272**			
Predictors	*Odds Ratios*	*CI*	*p*	*Predictors*	*Odds Ratios*	*CI*	*p*	*Predictors*	*Odds Ratios*	*CI*	*p*
(Intercept)	0.05	0.04 – 0.07	<0.001	(Intercept)	0	0.00 – 0.01	<0.001	(Intercept)	0.07	0.05 – 0.09	<0.001
Sex [Female]	0.7	0.68 – 0.73	<0.001	Sex [Female]	0.83	0.79 – 0.86	<0.001	Sex [Female]	0.78	0.74 – 0.83	<0.001
IMD Quintile [5]	(ref)			IMD Quintile [5]	(ref)			IMD Quintile [5]	(ref)		
IMD Quintile [4]	0.92	0.88 – 0.97	0.003	IMD Quintile [4]	0.89	0.84 – 0.94	<0.001	IMD Quintile [4]	0.95	0.88 – 1.04	0.258
IMD Quintile [3]	0.81	0.77 – 0.86	<0.001	IMD Quintile [3]	0.78	0.74 – 0.83	<0.001	IMD Quintile [3]	0.79	0.73 – 0.86	<0.001
IMD Quintile [2]	0.71	0.67 – 0.75	<0.001	IMD Quintile [2]	0.73	0.69 – 0.78	<0.001	IMD Quintile [2]	0.66	0.60 – 0.72	<0.001
IMD Quintile [1]	0.63	0.60 – 0.67	<0.001	IMD Quintile [1]	0.7	0.66 – 0.74	<0.001	IMD Quintile [1]	0.6	0.55 – 0.66	<0.001
IMD Quintile [Unknown]	0.38	0.27 – 0.52	<0.001	IMD Quintile [Unknown]	0.34	0.24 – 0.47	<0.001	IMD Quintile [Unknown]	0.67	0.47 – 0.95	0.028
Age	0.99	0.99 – 0.99	<0.001	Age	1.02	1.02 – 1.02	<0.001	Age	0.99	0.98 – 0.99	<0.001
Comorbidity score	0.95	0.94 – 0.95	<0.001	Comorbidity score	0.94	0.93 – 0.94	<0.001	Comorbidity score	0.94	0.93 – 0.95	<0.001
Ethnicity [White]	(ref)			Ethnicity [White]	(ref)			Ethnicity [White]	(ref)		
Ethnicity [Asian]	0.57	0.51 – 0.65	<0.001	Ethnicity [Asian]	0.66	0.58 – 0.74	<0.001	Ethnicity [Asian]	0.51	0.40 – 0.63	<0.001
Ethnicity [Black]	0.33	0.28 – 0.39	<0.001	Ethnicity [Black]	0.33	0.28 – 0.38	<0.001	Ethnicity [Black]	0.43	0.34 – 0.54	<0.001
Ethnicity [Mixed]	0.85	0.68 – 1.05	0.143	Ethnicity [Mixed]	0.54	0.43 – 0.68	<0.001	Ethnicity [Mixed]	0.89	0.62 – 1.25	0.522
Ethnicity [Other/Unknown]	1.85	1.77 – 1.93	<0.001	Ethnicity [Other/Unknown]	1.87	1.79 – 1.96	<0.001	Ethnicity [Other/Unknown]	2.16	2.02 – 2.32	<0.001
Year [2006]	(ref)			Year [2006]	(ref)			Year [2006]	(ref)		
Year [2007]	0.2	0.14 – 0.29	<0.001	Year [2007]	2.8	1.91 – 4.24	<0.001	Year [2007]	0.26	0.15 – 0.45	<0.001
Year [2008]	1.11	0.88 – 1.41	0.388	Year [2008]	3.82	2.64 – 5.71	<0.001	Year [2008]	0.7	0.46 – 1.06	0.091
Year [2009]	2.35	1.90 – 2.92	<0.001	Year [2009]	6.29	4.40 – 9.33	<0.001	Year [2009]	1.21	0.84 – 1.76	0.308
Year [2010]	4.48	3.68 – 5.51	<0.001	Year [2010]	12.13	8.57 – 17.84	<0.001	Year [2010]	3.28	2.39 – 4.60	<0.001
Year [2011]	6.19	5.10 – 7.58	<0.001	Year [2011]	16.45	11.66 – 24.13	<0.001	Year [2011]	5.46	4.03 – 7.59	<0.001
Year [2012]	6.92	5.71 – 8.47	<0.001	Year [2012]	20.57	14.61 – 30.13	<0.001	Year [2012]	6.19	4.58 – 8.58	<0.001
Year [2013]	8.13	6.71 – 9.93	<0.001	Year [2013]	22.23	15.80 – 32.55	<0.001	Year [2013]	7.67	5.69 – 10.61	<0.001
Year [2014]	8.28	6.84 – 10.11	<0.001	Year [2014]	25.82	18.37 – 37.76	<0.001	Year [2014]	8.76	6.50 – 12.09	<0.001
Year [2015]	10.05	8.31 – 12.27	<0.001	Year [2015]	25.74	18.31 – 37.65	<0.001	Year [2015]	11.22	8.35 – 15.48	<0.001
Year [2016]	10.7	8.86 – 13.07	<0.001	Year [2016]	29.45	20.96 – 43.05	<0.001	Year [2016]	12.87	9.57 – 17.77	<0.001
Year [2017]	11.85	9.81 – 14.47	<0.001	Year [2017]	30.45	21.68 – 44.50	<0.001	Year [2017]	14.68	10.92 – 20.24	<0.001
Year [2018]	12.65	10.47 – 15.44	<0.001	Year [2018]	36.4	25.93 – 53.18	<0.001	Year [2018]	14.1	10.48 – 19.46	<0.001
Year [2019]	12.17	10.07 – 14.86	<0.001	Year [2019]	37.26	26.54 – 54.45	<0.001	Year [2019]	14.73	10.94 – 20.34	<0.001

Supplementary Table 5: Coefficients from propensity score models (logistic regression models)

W391	THR (NEC) (org type=="ISHP")		
Predictors	*Odds Ratios*	*CI*	*p*
(Intercept)	0.04	0.03 – 0.06	<0.001
Sex [Female]	0.96	0.88 – 1.03	0.26
IMD Quintile [5]	(ref)		
IMD Quintile [4]	0.85	0.76 – 0.95	0.004
IMD Quintile [3]	0.73	0.65 – 0.82	<0.001
IMD Quintile [2]	0.53	0.46 – 0.59	<0.001
IMD Quintile [1]	0.43	0.37 – 0.49	<0.001
IMD Quintile [Unknown]	0.16	0.06 – 0.35	<0.001
Age	1	1.00 – 1.01	0.006
Comorbidity score	0.93	0.92 – 0.95	<0.001
Ethnicity [White]	(ref)		
Ethnicity [Asian]	0.44	0.19 – 0.85	0.027
Ethnicity [Black]	0.18	0.08 – 0.33	<0.001
Ethnicity [Mixed]	0.54	0.16 – 1.39	0.255
Ethnicity [Other/Unknown]	3.94	3.60 – 4.31	<0.001
Year [2006]	(ref)		
Year [2007]	1.64	1.19 – 2.29	0.003
Year [2008]	1.25	0.91 – 1.75	0.176
Year [2009]	2.67	1.97 – 3.67	<0.001
Year [2010]	7.46	5.62 – 10.09	<0.001
Year [2011]	10.52	7.93 – 14.21	<0.001
Year [2012]	9.23	6.93 – 12.52	<0.001
Year [2013]	7.22	5.39 – 9.84	<0.001
Year [2014]	1.82	1.28 – 2.61	0.001
Year [2015]	5.18	3.76 – 7.22	<0.001
Year [2016]	5.25	3.75 – 7.45	<0.001
Year [2017]	2.91	1.96 – 4.32	<0.001
Year [2018]	0.68	0.33 – 1.27	0.252
Year [2019]	2.3	1.48 – 3.54	<0.001

W401	TKR (cemented) (org type=="ISHP")		
Predictors	*Odds Ratios*	*CI*	*p*
(Intercept)	0.03	0.02 – 0.04	<0.001
Sex [Female]	0.94	0.91 – 0.97	<0.001
IMD Quintile [5]	(ref)		
IMD Quintile [4]	0.89	0.85 – 0.93	<0.001
IMD Quintile [3]	0.79	0.75 – 0.82	<0.001
IMD Quintile [2]	0.69	0.66 – 0.73	<0.001
IMD Quintile [1]	0.58	0.54 – 0.61	<0.001
IMD Quintile [Unknown]	0.23	0.18 – 0.29	<0.001
Age	1	1.00 – 1.00	0.033
Comorbidity score	0.94	0.93 – 0.94	<0.001
Ethnicity [White]	(ref)		
Ethnicity [Asian]	0.66	0.59 – 0.73	<0.001
Ethnicity [Black]	0.36	0.28 – 0.46	<0.001
Ethnicity [Mixed]	0.91	0.64 – 1.27	0.599
Ethnicity [Other/Unknown]	2.55	2.44 – 2.67	<0.001
Year [2006]	(ref)		
Year [2007]	1.94	1.49 – 2.57	<0.001
Year [2008]	2.72	2.11 – 3.57	<0.001
Year [2009]	4.24	3.30 – 5.51	<0.001
Year [2010]	9.07	7.15 – 11.68	<0.001
Year [2011]	10.73	8.47 – 13.80	<0.001
Year [2012]	12.37	9.78 – 15.90	<0.001
Year [2013]	14.93	11.82 – 19.16	<0.001
Year [2014]	16.89	13.39 – 21.67	<0.001
Year [2015]	20.02	15.87 – 25.67	<0.001
Year [2016]	22.19	17.60 – 28.44	<0.001
Year [2017]	23.1	18.32 – 29.60	<0.001
Year [2018]	26.56	21.07 – 34.04	<0.001
Year [2019]	27.3	21.66 – 34.98	<0.001

W411	TKR (no cement) (org type=="ISHP")		
Predictors	*Odds Ratios*	*CI*	*p*
(Intercept)	0.01	0.01 – 0.02	<0.001
Sex [Female]	0.99	0.93 – 1.04	0.6
IMD Quintile [5]	(ref)		
IMD Quintile [4]	0.92	0.85 – 0.99	0.03
IMD Quintile [3]	0.71	0.66 – 0.77	<0.001
IMD Quintile [2]	0.56	0.51 – 0.62	<0.001
IMD Quintile [1]	0.46	0.41 – 0.51	<0.001
IMD Quintile [Unknown]	0.16	0.08 – 0.29	<0.001
Age	1.01	1.01 – 1.01	<0.001
Comorbidity score	0.93	0.92 – 0.94	<0.001
Ethnicity [White]	(ref)		
Ethnicity [Asian]	0.47	0.37 – 0.58	<0.001
Ethnicity [Black]	0.28	0.18 – 0.42	<0.001
Ethnicity [Mixed]	0.62	0.34 – 1.06	0.096
Ethnicity [Other/Unknown]	1.6	1.48 – 1.73	<0.001
Year [2006]	(ref)		
Year [2007]	1.75	1.24 – 2.50	0.002
Year [2008]	1.26	0.88 – 1.84	0.214
Year [2009]	9.78	7.28 – 13.43	<0.001
Year [2010]	11.29	8.42 – 15.50	<0.001
Year [2011]	16.66	12.44 – 22.85	<0.001
Year [2012]	20.07	14.99 – 27.52	<0.001
Year [2013]	15.47	11.50 – 21.29	<0.001
Year [2014]	19.82	14.79 – 27.22	<0.001
Year [2015]	26.3	19.63 – 36.10	<0.001
Year [2016]	27.2	20.27 – 37.37	<0.001
Year [2017]	29.5	21.97 – 40.57	<0.001
Year [2018]	32.27	23.99 – 44.45	<0.001
Year [2019]	35.04	26.08 – 48.21	<0.001

Supplementary Table 5: Coefficients from propensity score models (logistic regression models)

Prostate resection (org type=="ISHP")

M653 *Predictors*	*Odds Ratios*	*CI*	*p*
(Intercept)	0.11	0.08 – 0.13	<0.001
Sex [Female]	3.56	2.03 – 5.99	<0.001
IMD Quintile [5]	(ref)		
IMD Quintile [4]	1.01	0.95 – 1.07	0.758
IMD Quintile [3]	0.96	0.91 – 1.02	0.212
IMD Quintile [2]	0.76	0.72 – 0.82	<0.001
IMD Quintile [1]	0.56	0.52 – 0.61	<0.001
IMD Quintile [Unknown]	0.32	0.20 – 0.48	<0.001
Age	0.97	0.97 – 0.98	<0.001
Comorbidity score	0.91	0.90 – 0.91	<0.001
Ethnicity [White]	(ref)		
Ethnicity [Asian]	0.71	0.63 – 0.81	<0.001
Ethnicity [Black]	0.28	0.21 – 0.38	<0.001
Ethnicity [Mixed]	1.2	0.84 – 1.66	0.295
Ethnicity [Other/Unknown]	2.87	2.73 – 3.02	<0.001
Year [2006]	(ref)		
Year [2007]	0.41	0.31 – 0.54	<0.001
Year [2008]	1.43	1.16 – 1.77	0.001
Year [2009]	2.1	1.72 – 2.58	<0.001
Year [2010]	3.4	2.82 – 4.13	<0.001
Year [2011]	4.49	3.74 – 5.43	<0.001
Year [2012]	6.02	5.03 – 7.25	<0.001
Year [2013]	6.89	5.76 – 8.30	<0.001
Year [2014]	7.18	6.01 – 8.64	<0.001
Year [2015]	7.86	6.58 – 9.46	<0.001
Year [2016]	8.3	6.95 – 10.00	<0.001
Year [2017]	8.2	6.86 – 9.87	<0.001
Year [2018]	8.81	7.37 – 10.62	<0.001
Year [2019]	10.04	8.41 – 12.07	<0.001

Hysterectomy (org type=="ISHP")

Q074 *Predictors*	*Odds Ratios*	*CI*	*p*
(Intercept)	0.02	0.01 – 0.04	<0.001
Sex [Female]	1.76	1.04 – 3.22	0.05
IMD Quintile [5]	(ref)		
IMD Quintile [4]	0.91	0.86 – 0.96	0.001
IMD Quintile [3]	0.87	0.83 – 0.92	<0.001
IMD Quintile [2]	0.74	0.70 – 0.78	<0.001
IMD Quintile [1]	0.6	0.57 – 0.64	<0.001
IMD Quintile [Unknown]	0.27	0.18 – 0.38	<0.001
Age	0.95	0.95 – 0.95	<0.001
Comorbidity score	0.81	0.81 – 0.82	<0.001
Ethnicity [White]	(ref)		
Ethnicity [Asian]	0.68	0.61 – 0.75	<0.001
Ethnicity [Black]	0.53	0.47 – 0.61	<0.001
Ethnicity [Mixed]	0.89	0.72 – 1.09	0.271
Ethnicity [Other/Unknown]	2.46	2.35 – 2.57	<0.001
Year [2006]	(ref)		
Year [2007]	0.3	0.17 – 0.52	<0.001
Year [2008]	2.82	2.03 – 3.98	<0.001
Year [2009]	7.01	5.19 – 9.72	<0.001
Year [2010]	13.59	10.14 – 18.71	<0.001
Year [2011]	20.39	15.26 – 28.02	<0.001
Year [2012]	22.47	16.82 – 30.85	<0.001
Year [2013]	27.18	20.36 – 37.30	<0.001
Year [2014]	28.74	21.54 – 39.42	<0.001
Year [2015]	32.58	24.44 – 44.67	<0.001
Year [2016]	35.25	26.44 – 48.33	<0.001
Year [2017]	33.07	24.80 – 45.35	<0.001
Year [2018]	32	23.99 – 43.91	<0.001
Year [2019]	32.79	24.58 – 44.97	<0.001

IH repair (prosthetics) (org type=="ISHP")

T212 *Predictors*	*Odds Ratios*	*CI*	*p*
(Intercept)	0.06	0.04 – 0.07	<0.001
Sex [Female]	0.92	0.82 – 1.05	0.218
IMD Quintile [5]	(ref)		
IMD Quintile [4]	0.92	0.86 – 0.98	0.014
IMD Quintile [3]	0.74	0.69 – 0.79	<0.001
IMD Quintile [2]	0.66	0.61 – 0.71	<0.001
IMD Quintile [1]	0.58	0.54 – 0.63	<0.001
IMD Quintile [Unknown]	0.38	0.24 – 0.56	<0.001
Age	0.99	0.99 – 0.99	<0.001
Comorbidity score	0.95	0.94 – 0.95	<0.001
Ethnicity [White]	(ref)		
Ethnicity [Asian]	0.79	0.67 – 0.93	0.004
Ethnicity [Black]	0.39	0.26 – 0.55	<0.001
Ethnicity [Mixed]	0.92	0.59 – 1.38	0.685
Ethnicity [Other/Unknown]	1.97	1.86 – 2.10	<0.001
Year [2006]	(ref)		
Year [2007]	0.49	0.33 – 0.74	0.001
Year [2008]	1.12	0.81 – 1.57	0.5
Year [2009]	2.45	1.83 – 3.34	<0.001
Year [2010]	5.55	4.23 – 7.42	<0.001
Year [2011]	7.76	5.95 – 10.33	<0.001
Year [2012]	9.44	7.25 – 12.55	<0.001
Year [2013]	10.46	8.04 – 13.88	<0.001
Year [2014]	11.72	9.02 – 15.53	<0.001
Year [2015]	12.88	9.91 – 17.07	<0.001
Year [2016]	14	10.77 – 18.56	<0.001
Year [2017]	15.09	11.62 – 19.99	<0.001
Year [2018]	15.4	11.86 – 20.41	<0.001
Year [2019]	16.81	12.94 – 22.28	<0.001

Supplementary Table 5: Coefficients from propensity score models (logistic regression models)

**V255**	**Lumbar decompression (org type=="ISHP")**			**W371**	**THR (cemented) (org type=="ISHP")**			**W381**	**THR (no cement) (org type=="ISHP")**		
Predictors	*Odds Ratios*	*CI*	*p*	*Predictors*	*Odds Ratios*	*CI*	*p*	*Predictors*	*Odds Ratios*	*CI*	*p*
(Intercept)	0.04	0.03 – 0.07	<0.001	(Intercept)	0.1	0.08 – 0.11	<0.001	(Intercept)	0.01	0.00 – 0.01	<0.001
Sex [Female]	0.99	0.96 – 1.03	0.77	Sex [Female]	1.05	1.01 – 1.08	0.009	Sex [Female]	1.04	1.00 – 1.07	0.029
IMD Quintile [5]	(ref)			IMD Quintile [5]	(ref)			IMD Quintile [5]	(ref)		
IMD Quintile [4]	1.01	0.95 – 1.07	0.761	IMD Quintile [4]	0.86	0.83 – 0.90	<0.001	IMD Quintile [4]	1.03	0.98 – 1.08	0.195
IMD Quintile [3]	0.96	0.90 – 1.02	0.148	IMD Quintile [3]	0.74	0.71 – 0.77	<0.001	IMD Quintile [3]	0.92	0.87 – 0.96	0.001
IMD Quintile [2]	0.85	0.80 – 0.90	<0.001	IMD Quintile [2]	0.63	0.60 – 0.66	<0.001	IMD Quintile [2]	0.74	0.70 – 0.78	<0.001
IMD Quintile [1]	0.7	0.66 – 0.75	<0.001	IMD Quintile [1]	0.49	0.46 – 0.52	<0.001	IMD Quintile [1]	0.6	0.56 – 0.64	<0.001
IMD Quintile [Unknown]	0.07	0.05 – 0.11	<0.001	IMD Quintile [Unknown]	0.22	0.16 – 0.29	<0.001	IMD Quintile [Unknown]	0.08	0.06 – 0.10	<0.001
Age	0.99	0.99 – 0.99	<0.001	Age	0.99	0.99 – 0.99	<0.001	Age	1.01	1.01 – 1.02	<0.001
Comorbidity score	0.94	0.94 – 0.95	<0.001	Comorbidity score	0.91	0.91 – 0.92	<0.001	Comorbidity score	0.93	0.93 – 0.94	<0.001
Ethnicity [White]	(ref)			Ethnicity [White]	(ref)			Ethnicity [White]	(ref)		
Ethnicity [Asian]	0.81	0.71 – 0.92	0.002	Ethnicity [Asian]	0.61	0.45 – 0.83	0.002	Ethnicity [Asian]	0.53	0.40 – 0.69	<0.001
Ethnicity [Black]	0.56	0.43 – 0.70	<0.001	Ethnicity [Black]	0.55	0.38 – 0.77	0.001	Ethnicity [Black]	0.36	0.28 – 0.46	<0.001
Ethnicity [Mixed]	0.74	0.53 – 1.00	0.059	Ethnicity [Mixed]	0.6	0.33 – 1.01	0.071	Ethnicity [Mixed]	0.5	0.32 – 0.74	0.001
Ethnicity [Other/Unknown]	1.7	1.62 – 1.78	<0.001	Ethnicity [Other/Unknown]	3.25	3.12 – 3.39	<0.001	Ethnicity [Other/Unknown]	1.81	1.73 – 1.89	<0.001
Year [2006]	(ref)			Year [2006]	(ref)			Year [2006]	(ref)		
Year [2007]	1.41	0.84 – 2.45	0.208	Year [2007]	1.23	1.05 – 1.44	0.01	Year [2007]	2.29	1.54 – 3.50	<0.001
Year [2008]	1.37	0.84 – 2.36	0.229	Year [2008]	3.07	2.68 – 3.54	<0.001	Year [2008]	5.15	3.58 – 7.69	<0.001
Year [2009]	2.74	1.73 – 4.59	<0.001	Year [2009]	3.11	2.70 – 3.58	<0.001	Year [2009]	10.95	7.70 – 16.20	<0.001
Year [2010]	3.73	2.39 – 6.18	<0.001	Year [2010]	4.12	3.59 – 4.73	<0.001	Year [2010]	17.42	12.30 – 25.69	<0.001
Year [2011]	6.83	4.42 – 11.24	<0.001	Year [2011]	5.2	4.55 – 5.96	<0.001	Year [2011]	23.31	16.48 – 34.36	<0.001
Year [2012]	8.93	5.80 – 14.65	<0.001	Year [2012]	5.52	4.84 – 6.33	<0.001	Year [2012]	24.51	17.34 – 36.11	<0.001
Year [2013]	10.63	6.92 – 17.43	<0.001	Year [2013]	6.66	5.84 – 7.61	<0.001	Year [2013]	30.14	21.32 – 44.39	<0.001
Year [2014]	12.85	8.37 – 21.06	<0.001	Year [2014]	6.77	5.95 – 7.75	<0.001	Year [2014]	34.79	24.63 – 51.20	<0.001
Year [2015]	14.16	9.23 – 23.20	<0.001	Year [2015]	7.46	6.55 – 8.54	<0.001	Year [2015]	41.56	29.43 – 61.17	<0.001
Year [2016]	16.15	10.52 – 26.45	<0.001	Year [2016]	7.05	6.18 – 8.07	<0.001	Year [2016]	43.3	30.66 – 63.72	<0.001
Year [2017]	17.32	11.29 – 28.37	<0.001	Year [2017]	7.07	6.19 – 8.10	<0.001	Year [2017]	54.74	38.77 – 80.55	<0.001
Year [2018]	16.39	10.68 – 26.83	<0.001	Year [2018]	8.07	7.07 – 9.24	<0.001	Year [2018]	63.16	44.73 – 92.94	<0.001
Year [2019]	18.2	11.87 – 29.80	<0.001	Year [2019]	8.3	7.28 – 9.51	<0.001	Year [2019]	65.65	46.49 – 96.61	<0.001

Supplementary Table 5: Coefficients from propensity score models (logistic regression models)

	**TKR (NEC)**				**THR (cemented acetabulum)**				**THR (cemented femoral stem)**		
**W421**	**(org type=="ISHP")**			**W931**	**(org type=="ISHP")**			**W941**	**(org type=="ISHP")**		
Predictors	*Odds Ratios*	*CI*	*p*	*Predictors*	*Odds Ratios*	*CI*	*p*	*Predictors*	*Odds Ratios*	*CI*	*p*
(Intercept)	0.21	0.16 – 0.27	<0.001	(Intercept)	0.01	0.00 – 0.04	<0.001	(Intercept)	0.01	0.00 – 0.01	<0.001
Sex [Female]	0.95	0.90 – 1.00	0.074	Sex [Female]	1.08	1.00 – 1.16	0.064	Sex [Female]	1.02	0.98 – 1.05	0.282
IMD Quintile [5]	(ref)			IMD Quintile [5]	(ref)			IMD Quintile [5]	(ref)		
IMD Quintile [4]	0.99	0.91 – 1.08	0.804	IMD Quintile [4]	0.77	0.69 – 0.86	<0.001	IMD Quintile [4]	1	0.96 – 1.05	0.932
IMD Quintile [3]	0.8	0.73 – 0.87	<0.001	IMD Quintile [3]	0.59	0.53 – 0.66	<0.001	IMD Quintile [3]	0.99	0.95 – 1.04	0.7
IMD Quintile [2]	0.57	0.52 – 0.62	<0.001	IMD Quintile [2]	0.52	0.46 – 0.59	<0.001	IMD Quintile [2]	0.89	0.84 – 0.93	<0.001
IMD Quintile [1]	0.41	0.37 – 0.45	<0.001	IMD Quintile [1]	0.37	0.32 – 0.41	<0.001	IMD Quintile [1]	0.65	0.61 – 0.69	<0.001
IMD Quintile [Unknown]	0.93	0.60 – 1.42	0.726	IMD Quintile [Unknown]	1.7	1.27 – 2.27	<0.001	IMD Quintile [Unknown]	0.1	0.07 – 0.14	<0.001
Age	1.01	1.00 – 1.01	0.001	Age	1	1.00 – 1.01	0.078	Age	1	1.00 – 1.01	<0.001
Comorbidity score	0.88	0.87 – 0.89	<0.001	Comorbidity score	0.93	0.92 – 0.94	<0.001	Comorbidity score	0.93	0.93 – 0.94	<0.001
Ethnicity [White]	(ref)			Ethnicity [White]	(ref)			Ethnicity [White]	(ref)		
Ethnicity [Asian]	0.36	0.28 – 0.45	<0.001	Ethnicity [Asian]	0.61	0.31 – 1.10	0.117	Ethnicity [Asian]	0.51	0.37 – 0.69	<0.001
Ethnicity [Black]	0.15	0.10 – 0.21	<0.001	Ethnicity [Black]	0.4	0.15 – 0.89	0.04	Ethnicity [Black]	0.57	0.41 – 0.78	0.001
Ethnicity [Mixed]	0.33	0.15 – 0.65	0.003	Ethnicity [Mixed]	0.82	0.26 – 2.17	0.706	Ethnicity [Mixed]	0.73	0.46 – 1.12	0.169
Ethnicity [Other/Unknown]	3.7	3.46 – 3.95	<0.001	Ethnicity [Other/Unknown]	3.12	2.80 – 3.48	<0.001	Ethnicity [Other/Unknown]	1.68	1.61 – 1.75	<0.001
Year [2006]	(ref)			Year [2006]	(ref)			Year [2006]	(ref)		
Year [2007]	0.54	0.46 – 0.64	<0.001	Year [2007]	0.67	0.15 – 3.46	0.608	Year [2007]	2.2	1.23 – 4.24	0.012
Year [2008]	2.37	2.07 – 2.73	<0.001	Year [2008]	3.09	1.08 – 13.04	0.066	Year [2008]	0.49	0.22 – 1.08	0.074
Year [2009]	3.05	2.66 – 3.51	<0.001	Year [2009]	2.78	0.98 – 11.65	0.094	Year [2009]	3.05	1.75 – 5.75	<0.001
Year [2010]	2.99	2.59 – 3.46	<0.001	Year [2010]	7.89	2.89 – 32.50	0.001	Year [2010]	9.03	5.38 – 16.66	<0.001
Year [2011]	3.95	3.42 – 4.57	<0.001	Year [2011]	10.95	4.06 – 44.89	<0.001	Year [2011]	10.8	6.45 – 19.85	<0.001
Year [2012]	2.74	2.35 – 3.21	<0.001	Year [2012]	15.84	5.90 – 64.81	<0.001	Year [2012]	15.78	9.47 – 28.94	<0.001
Year [2013]	2.25	1.90 – 2.66	<0.001	Year [2013]	27.03	10.11 – 110.36	<0.001	Year [2013]	23.39	14.08 – 42.79	<0.001
Year [2014]	1.98	1.65 – 2.38	<0.001	Year [2014]	30.16	11.30 – 123.01	<0.001	Year [2014]	33.31	20.09 – 60.85	<0.001
Year [2015]	3.78	3.17 – 4.51	<0.001	Year [2015]	36.32	13.63 – 148.03	<0.001	Year [2015]	38.65	23.33 – 70.59	<0.001
Year [2016]	3.55	2.94 – 4.29	<0.001	Year [2016]	45.93	17.25 – 187.12	<0.001	Year [2016]	47.1	28.45 – 85.96	<0.001
Year [2017]	3.68	3.01 – 4.51	<0.001	Year [2017]	55.61	20.88 – 226.54	<0.001	Year [2017]	49.95	30.18 – 91.16	<0.001
Year [2018]	1.06	0.81 – 1.39	0.654	Year [2018]	65.12	24.45 – 265.33	<0.001	Year [2018]	54.97	33.22 – 100.31	<0.001
Year [2019]	1.39	1.06 – 1.81	0.015	Year [2019]	89.44	33.55 – 364.58	<0.001	Year [2019]	58.1	35.12 – 106.01	<0.001
