# Supplementary material for: Outcomes for surgical procedures funded by the English health service but carried out in public versus independent hospitals: a database study
Source: BMJ Qual Saf. 2021 Sep 7;31(7):515–25. doi: 10.1136/bmjqs-2021-013522 (PMC9234423; doi:10.1136/bmjqs-2021-013522)
Supplement: Supplementary data [file bmjqs-2021-013522supp008.pdf]

Supplementary Table 6: Standardised mean difference (SMD) before and after propensity score matching. Values of SMD > 0.1 are highlighted

Covariate	Operation type	SMD before	SMD after	Operation type	SMD before	SMD after	Operation type	SMD before	SMD after
Sex	F091	0.046	0.001	T242	**0.184**	0.006	W391	0.016	0.003
Operation year		**0.802**	0.034		**0.679**	0.044		**0.722**	0.066
Deprivation quintile		0.083	0.009		**0.189**	0.016		**0.329**	0.024
Age		**0.177**	0.019		0.074	0.011		0.025	0.009
Charlson comorbidity score		0.053	0.001		**0.127**	0.003		**0.219**	0.027
Ethnicity		**0.171**	0.020		**0.334**	0.037		**0.568**	0.012
Sex	F093	0.090	0.000	T243	**0.188**	0.013	W401	0.040	0.008
Operation year		**0.726**	0.023		**0.647**	0.037		**0.685**	0.032
Deprivation quintile		**0.106**	0.014		**0.188**	0.012		**0.225**	0.018
Age		**0.158**	0.005		**0.382**	0.005		0.053	0.003
Charlson comorbidity score		0.032	0.008		0.050	0.002		**0.195**	0.003
Ethnicity		**0.157**	0.008		**0.368**	0.041		**0.360**	0.022
Sex	J183	**0.141**	0.004	T272	**0.119**	0.003	W411	0.034	0.006
Operation year		**0.566**	0.026		**0.819**	0.050		**0.862**	0.034
Deprivation quintile		**0.184**	0.015		**0.190**	0.020		**0.297**	0.021
Age		**0.152**	0.000		**0.214**	0.009		0.055	0.009
Charlson comorbidity score		**0.153**	0.001		**0.171**	0.004		**0.166**	0.013
Ethnicity		**0.313**	0.019		**0.358**	0.043		**0.203**	0.022
Sex	M653	0.027	0.005	V255	0.016	0.000	W421	0.048	0.038
Operation year		**0.663**	0.055		**0.565**	0.032		**0.494**	0.085
Deprivation quintile		**0.225**	0.014		**0.226**	0.019		**0.366**	0.042
Age		**0.306**	0.013		**0.245**	0.005		0.041	0.012
Charlson comorbidity score		**0.431**	0.012		**0.195**	0.004		**0.341**	0.011
Ethnicity		**0.416**	0.036		**0.263**	0.017		**0.653**	0.032
Sex	Q074	0.001	0.009	W371	0.021	0.008	W931	0.007	0.017
Operation year		**0.699**	0.061		**0.459**	0.036		**0.853**	0.071
Deprivation quintile		**0.140**	0.039		**0.250**	0.012		**0.332**	0.042
Age		**0.691**	0.003		**0.114**	0.009		0.026	0.006
Charlson comorbidity score		**0.734**	0.037		**0.310**	0.007		**0.233**	0.010
Ethnicity		**0.291**	0.058		**0.412**	0.012		**0.388**	0.099
Sex	T212	0.011	0.005	W381	0.011	0.009	W941	0.007	0.001
Operation year		**0.750**	0.034		**0.726**	0.039		**0.709**	0.039
Deprivation quintile		**0.168**	0.015		**0.286**	0.009		**0.185**	0.011
Age		**0.201**	0.003		**0.105**	0.002		0.002	0.013
Charlson comorbidity score		**0.170**	0.006		**0.160**	0.003		**0.217**	0.005
Ethnicity		**0.285**	0.046		**0.259**	0.034		**0.248**	0.025
