# Supplementary material for: Outcomes for surgical procedures funded by the English health service but carried out in public versus independent hospitals: a database study
Source: BMJ Qual Saf. 2021 Sep 7;31(7):515–25. doi: 10.1136/bmjqs-2021-013522 (PMC9234423; doi:10.1136/bmjqs-2021-013522)

Supplementary Figure 2: Histograms showing propensity score distribution before and after matching. Blue-green histograms are for operations taking place in NHS hospitals where as red histograms show the distribution of propensity scores for operations taking place in ISHPs.

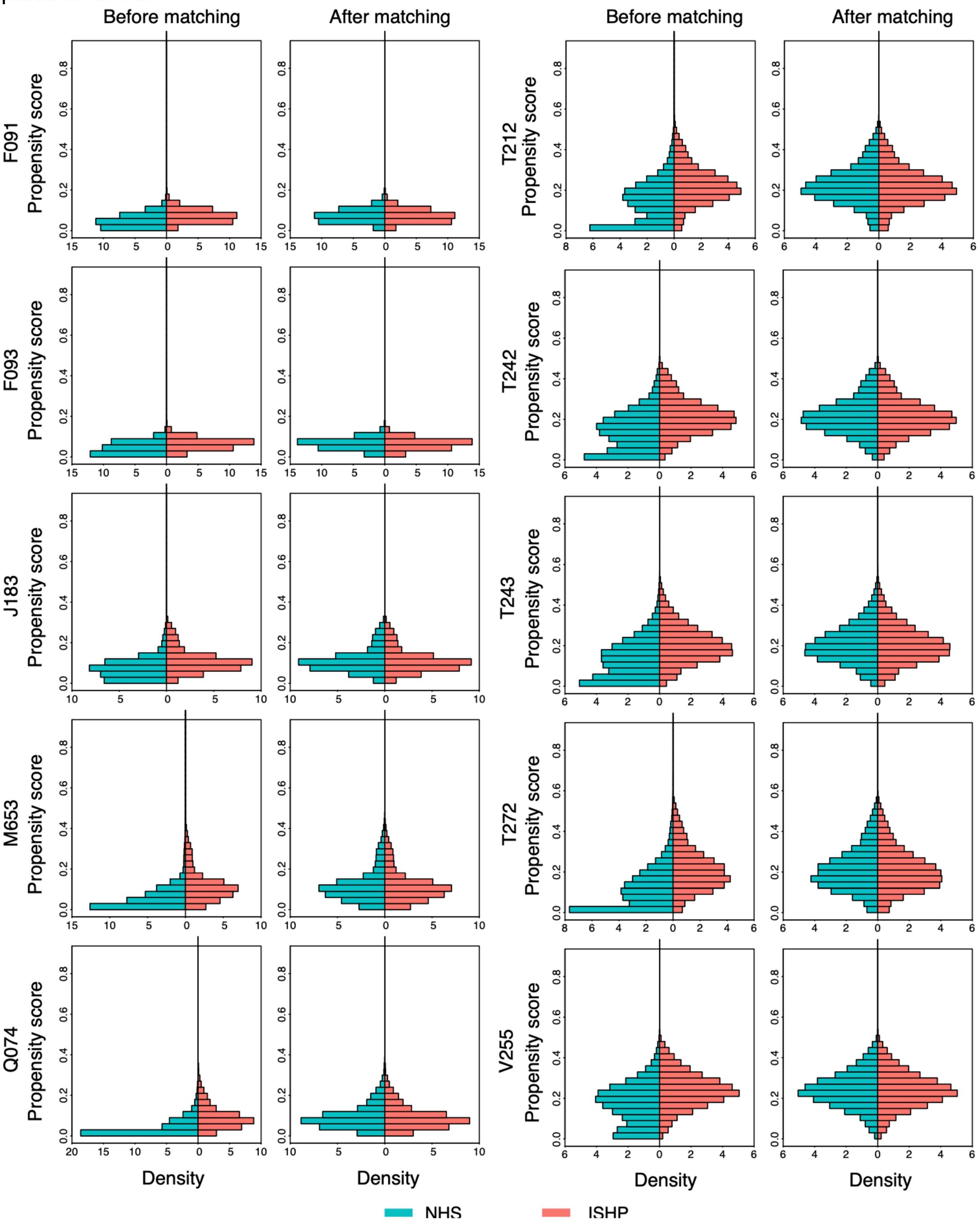

Supplementary Figure 2 cont.

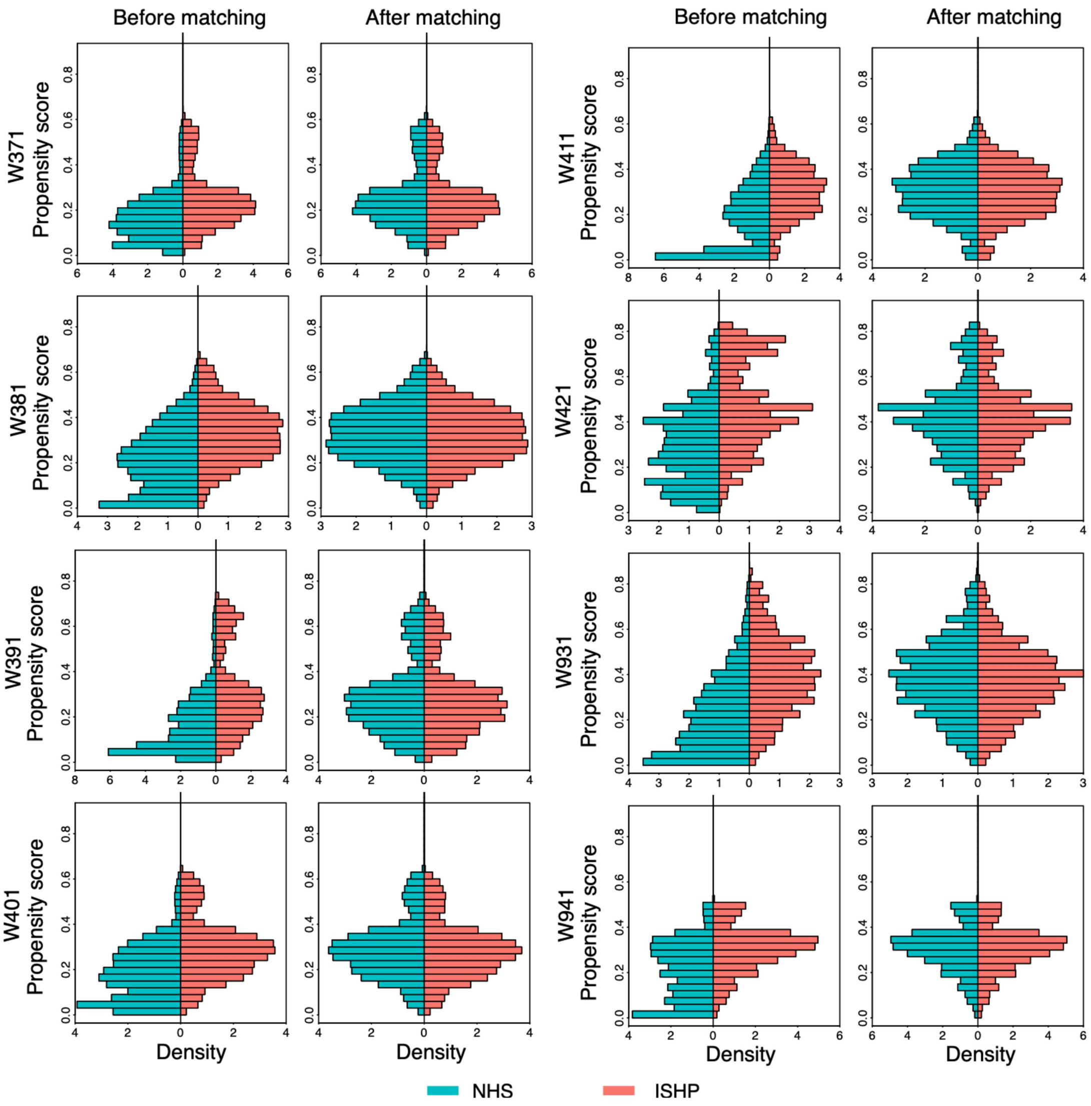

Supplement: Supplementary data [file bmjqs-2021-013522supp009.pdf]
