# Supplementary material for: Outcomes for surgical procedures funded by the English health service but carried out in public versus independent hospitals: a database study
Source: BMJ Qual Saf. 2021 Sep 7;31(7):515–25. doi: 10.1136/bmjqs-2021-013522 (PMC9234423; doi:10.1136/bmjqs-2021-013522)
Supplement: Supplementary data [file bmjqs-2021-013522supp010.pdf]

Supplementary Table 7: Event counts by operation type in the matched data

		In-hospital death (A)		Emergency transfer (B1)		Non-emergency transfer (B2)		Long term stay (C)		Death (D)**		All cause readmission (E)**		Within-specialty readmission (E1)**	
Operation type	Total*	NHS	ISHP	NHS	ISHP	NHS	ISHP	NHS	ISHP	NHS	ISHP	NHS	ISHP	NHS	ISHP
Wisdom tooth impacted	17184	0	≤7+	≤7	0	≤7	0	0	0	≤7	0	179	128	79	42
Wisdom tooth NEC	17626	0	0	0	≤7	11	≤7	0	0	0	0	195	143	41	21
Cholecystectomy++	20052	≤7	0	11	11	24	≤7	≤7	0	≤7	≤7	1393	1070	1072	815
Prostate resection	10184	≤7	0	≤7	9	11	≤7	≤7	0	9	≤7	822	525	425	253
Hysterectomy	13674	≤7	0	≤7	≤7	≤7	≤7	0	0	0	≤7	1233	833	967	580
IH repair (prosthetics)	8781	0	0	≤7	≤7	≤7	≤7	0	0	≤7	≤7	405	237	248	121
UH repair (prosthetics)	17296	≤7	0	0	≤7	11	≤7	≤7	0	≤7	≤7	809	356	611	232
UH repair (sutures)	13970	≤7	0	≤7	0	≤7	≤7	0	0	≤7	≤7	513	254	358	149
VH repair (prosthetics)	6322	≤7	≤7	≤7	≤7	≤7	≤7	≤7	0	≤7	≤7	358	147	251	96
Lumbar decompression	13770	≤7	≤7	11	≤7	50	≤7	≤7	≤7	≤7	≤7	595	317	325	119
THR (cemented)++	19975	22	≤7	32	46	270	24	9	≤7	17	15	1076	888	411	307
THR (no cement)++	19950	8	0	21	11	122	13	11	≤7	8	13	977	685	317	211
THR (NEC)	3355	≤7	0	≤7	≤7	84	0	8	≤7	≤7	≤7	201	131	65	36
TKR (cemented)++	19837	13	≤7	33	38	127	12	≤7	0	11	13	1116	818	420	208
TKR (no cement)	6979	≤7	≤7	12	21	72	≤7	≤7	≤7	≤7	≤7	423	293	137	63
TKR (NEC)	7738	10	≤7	≤7	16	144	10	≤7	≤7	8	16	489	352	181	90
THR (cemented acetabulum)	4326	≤7	0	≤7	≤7	27	≤7	0	0	≤7	≤7	249	171	64	39
THR (cemented femoral stem)++	19968	15	≤7	35	39	156	14	≤7	0	9	13	1015	725	362	187

** Number of operations in each of the matched NHS and ISHP groups*
*** D-E are events occurring within 28 days of discharge. Note that emergency readmission and death may both occur*
+ Small numbers ≤7 have been censored for non-identifiability purposes.
++ The raw data set for these operation types was randomly subsampled so as to reduce the computational expense of the matching process.
