# Supplementary material for: Outcomes for surgical procedures funded by the English health service but carried out in public versus independent hospitals: a database study
Source: BMJ Qual Saf. 2021 Sep 7;31(7):515–25. doi: 10.1136/bmjqs-2021-013522 (PMC9234423; doi:10.1136/bmjqs-2021-013522)

Supplementary Figure 3. Cumulative incidence of events by operation type and provider type (NHS vs ISHP). First and third columns: Cumulative incidence of all in-hospital events post-operation. Second and fourth columns: Cumulative incidence of post-discharge events. Events occurring within the same time frame (in hospital or post-discharge) are considered as competing risks.

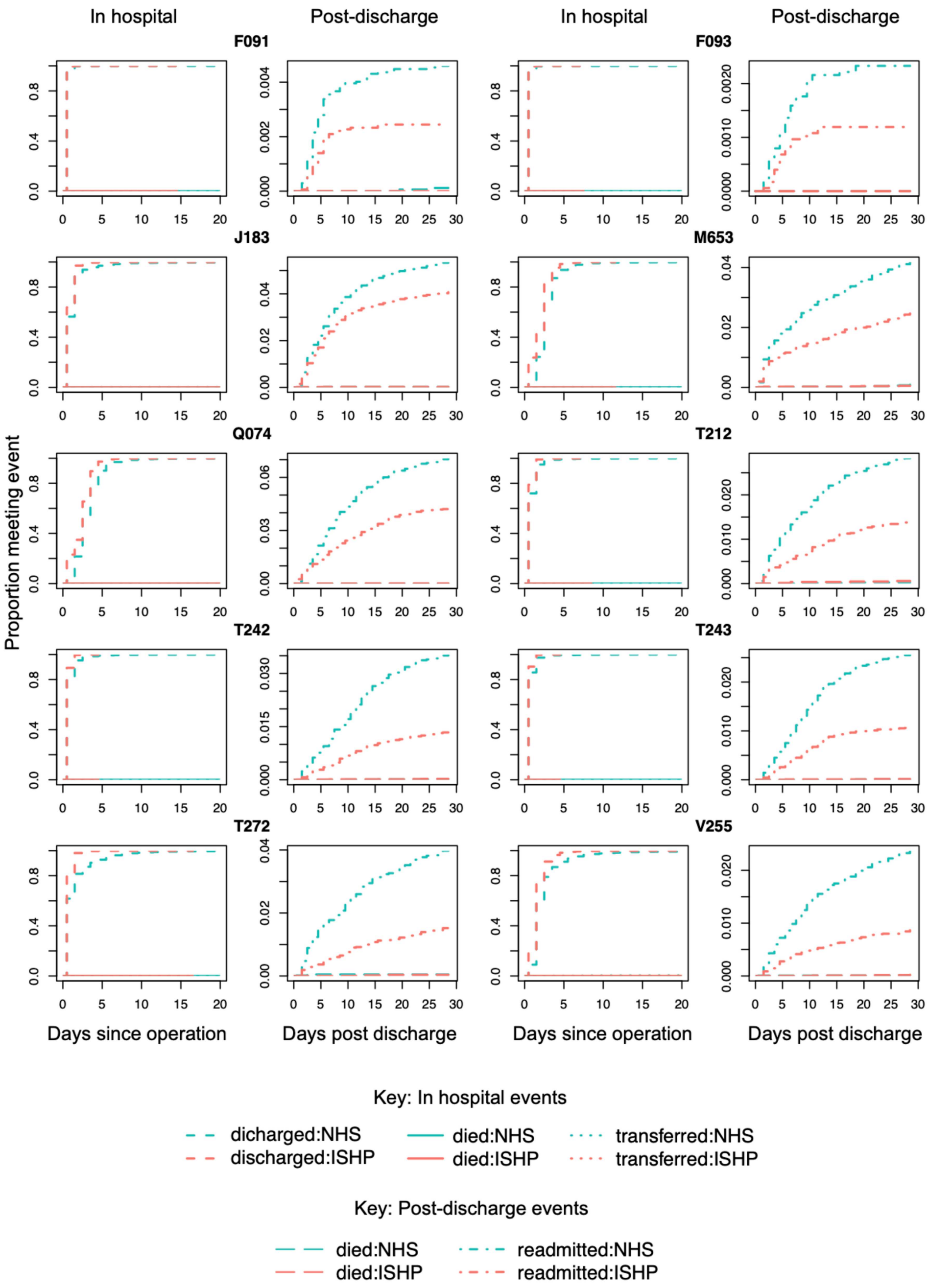

Supplementary Figure 3 cont.

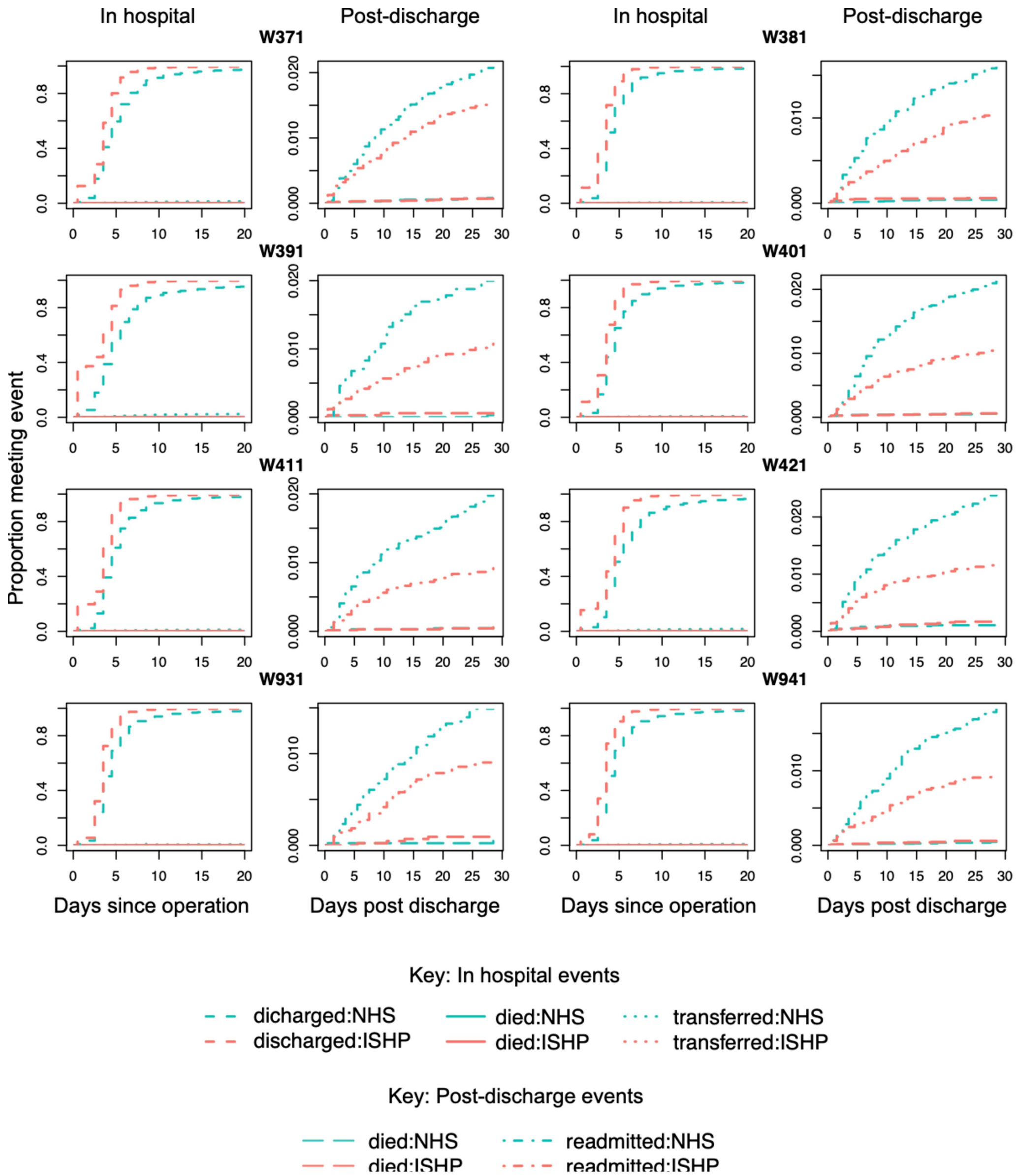

Supplement: Supplementary data [file bmjqs-2021-013522supp011.pdf]
