# Supplementary material for: Outcomes for surgical procedures funded by the English health service but carried out in public versus independent hospitals: a database study
Source: BMJ Qual Saf. 2021 Sep 7;31(7):515–25. doi: 10.1136/bmjqs-2021-013522 (PMC9234423; doi:10.1136/bmjqs-2021-013522)
Supplement: Supplementary data [file bmjqs-2021-013522supp012.pdf]

Supplementary Table 8: Hazard ratios for all in-hospital outcomes (discharge, in-hospital death, between-hospital transfer). Results highlighted in bold are significant at the 95% level. The * indicates hazard ratios that could not be reliably estimated because there were zero events for one or both of the provider types.

	Hazard ratio (95% CI) for ISHP vs NHS hospital			
**Operation**	**Discharge**	**Death**	**Emergency transfer**	**Other transfer**
Wisdom tooth impacted	**1.07 (1.05,1.09)**	*	*	*
Wisdom tooth NEC	**1.08 (1.06,1.10)**	*	*	0.09 (0.01,1.04)
Cholecystectomy	**1.34 (1.25,1.45)**	*	2.07 (0.70,6.13)	0.57 (0.18,1.77)
Prostate resection	**1.59 (1.40,1.81)**	*	**17.57 (2.79,110.56)**	0.35 (0.07,1.73)
Hysterectomy	**1.53 (1.36,1.73)**	*	3.03 (0.53,17.53)	1.14 (0.26,4.96)
IH repair (prosthetics)	**1.24 (1.17,1.32)**	*	0.78 (0.02,26.42)	0.28 (0.03,2.66)
UH repair (prosthetics)	**1.32 (1.26,1.39)**	*	*	**0.11 (0.01,0.87)**
UH repair (sutures)	**1.18 (1.11,1.25)**	*	*	3.48 (0.59,20.59)
VH repair (prosthetics)	**1.82 (1.70,1.96)**	1.41 (0.14,13.90)	1.29 (0.06,27.22)	0.49 (0.05,5.30)
Lumbar decompression	**1.54 (1.35,1.77)**	1.15 (0.08,16.92)	2.15 (0.87,5.30)	**0.19 (0.06,0.65)**
THR (cemented)	**1.81 (1.61,2.04)**	*	**3.14 (1.57,6.27)**	**0.26 (0.15,0.46)**
THR (no cement)	**1.79 (1.61,1.98)**	*	0.83 (0.31,2.18)	**0.32 (0.14,0.70)**
THR (NEC)	**2.15 (1.72,2.68)**	*	1.79 (0.54,5.93)	*
TKR (cemented)	**1.90 (1.73,2.09)**	0.26 (0.03,1.93)	**2.23 (1.06,4.69)**	**0.32 (0.16,0.67)**
TKR (no cement)	**1.95 (1.65,2.29)**	3.32 (0.47,23.36)	**3.20 (1.46,6.99)**	**0.18 (0.06,0.54)**
TKR (NEC)	**1.99 (1.72,2.29)**	0.22 (0.03,1.74)	**4.56 (1.35,15.33)**	**0.19 (0.08,0.45)**
THR (cemented acetabulum)	**1.75 (1.40,2.19)**	*	1.24 (0.30,5.13)	1.14 (0.35,3.71)
THR (cemented femoral stem)	**1.88 (1.69,2.10)**	**0.15 (0.02,0.94)**	**2.48 (1.11,5.53)**	**0.27 (0.12,0.63)**
