# Supplementary material for: Outcomes for surgical procedures funded by the English health service but carried out in public versus independent hospitals: a database study
Source: BMJ Qual Saf. 2021 Sep 7;31(7):515–25. doi: 10.1136/bmjqs-2021-013522 (PMC9234423; doi:10.1136/bmjqs-2021-013522)
Supplement: Supplementary data [file bmjqs-2021-013522supp013.pdf]

Supplementary Table 9: Hazard ratios for all outcomes post-discharge and within 28 days (readmission, death). Results highlighted in bold are significant at the 95% level. The * indicates hazard ratios that could not be reliably estimated because there were zero events for one or both of the provider types.

	Hazard ratio (95% CI) for ISHP vs NHS hospital		
**Operation**	**Within specialty readmission**	**All cause readmission**	**Death**
Wisdom tooth impacted	**0.53 (0.31,0.91)**	0.71 (0.51,1.00)	*
Wisdom tooth NEC	**0.51 (0.29,0.91)**	**0.73 (0.54,1.00)**	*
Cholecystectomy	**0.75 (0.67,0.85)**	**0.76 (0.69,0.84)**	1.30 (0.36,4.73)
Prostate resection	**0.59 (0.45,0.77)**	**0.63 (0.55,0.72)**	0.67 (0.23,1.97)
Hysterectomy	**0.59 (0.51,0.68)**	**0.66 (0.59,0.75)**	*
IH repair (prosthetics)	**0.48 (0.39,0.60)**	**0.58 (0.49,0.68)**	1.60 (0.33,7.76)
UH repair (prosthetics)	**0.38 (0.30,0.46)**	**0.43 (0.37,0.51)**	2.07 (0.36,12.05)
UH repair (sutures)	**0.41 (0.32,0.53)**	**0.49 (0.41,0.59)**	1.12 (0.16,7.96)
VH repair (prosthetics)	**0.38 (0.29,0.49)**	**0.40 (0.34,0.48)**	0.63 (0.12,3.42)
Lumbar decompression	**0.36 (0.28,0.46)**	**0.53 (0.45,0.62)**	1.96 (0.37,10.51)
THR (cemented)	**0.74 (0.61,0.88)**	**0.81 (0.72,0.92)**	0.90 (0.46,1.77)
THR (no cement)	**0.66 (0.54,0.81)**	**0.69 (0.62,0.77)**	1.80 (0.67,4.79)
THR (NEC)	**0.54 (0.35,0.83)**	**0.63 (0.49,0.80)**	2.08 (0.18,24.45)
TKR (cemented)	**0.49 (0.40,0.59)**	**0.72 (0.66,0.80)**	1.17 (0.53,2.58)
TKR (no cement)	**0.45 (0.33,0.62)**	**0.68 (0.57,0.81)**	1.47 (0.30,7.16)
TKR (NEC)	**0.49 (0.37,0.64)**	**0.70 (0.59,0.84)**	1.85 (0.88,3.86)
THR (cemented acetabulum)	**0.60 (0.37,0.99)**	**0.68 (0.51,0.91)**	2.95 (0.87,9.97)
THR (cemented femoral stem)	**0.51 (0.41,0.63)**	**0.71 (0.62,0.81)**	1.63 (0.75,3.53)
