# Supplementary material for: Outcomes for surgical procedures funded by the English health service but carried out in public versus independent hospitals: a database study
Source: BMJ Qual Saf. 2021 Sep 7;31(7):515–25. doi: 10.1136/bmjqs-2021-013522 (PMC9234423; doi:10.1136/bmjqs-2021-013522)
Supplement: Supplementary data [file bmjqs-2021-013522supp015.pdf]

Supplementary Table 10: Hazard ratios for all in-hospital outcomes (discharge, in-hospital death, between-hospital transfer), comparing NHS hospitals with for-profit ISHPs. Results highlighted in bold are significant at the 95% level. The * indicates hazard ratios that could not be reliably estimated because there were zero events for one or both of the provider types.

	Hazard ratio (95% CI) for for-profit ISHP vs NHS hospital			
**Operation**	**Discharge**	**Death**	**Emergency transfer**	**Other transfer**
Wisdom tooth impacted	**1.07 (1.05,1.09)**	*	*	*
Wisdom tooth NEC	**1.08 (1.06,1.09)**	*	*	0.14 (0.01,1.72)
Cholecystectomy	**1.37 (1.26,1.48)**	*	0.69 (0.16,2.97)	0.30 (0.06,1.50)
Prostate resection	**1.65 (1.43,1.90)**	*	**4.69 (1.37,16.01)**	0.54 (0.10,2.97)
Hysterectomy	**1.58 (1.39,1.80)**	*	5.45 (0.89,33.39)	0.40 (0.09,1.81)
IH repair (prosthetics)	**1.28 (1.20,1.37)**	*	*	0.91 (0.07,11.55)
UH repair (prosthetics)	**1.36 (1.28,1.43)**	*	**9.38 (1.77,49.58)**	0.17 (0.02,1.45)
UH repair (sutures)	**1.20 (1.12,1.28)**	*	*	0.33 (0.03,3.15)
VH repair (prosthetics)	**1.89 (1.75,2.05)**	*	*	0.25 (0.03,2.21)
Lumbar decompression	**1.51 (1.30,1.75)**	0.76 (0.09,6.64)	0.49 (0.10,2.46)	**0.08 (0.01,0.59)**
THR (cemented)	**1.84 (1.63,2.08)**	**0.16 (0.04,0.67)**	1.17 (0.47,2.92)	**0.24 (0.11,0.53)**
THR (no cement)	**1.78 (1.58,1.99)**	*	0.66 (0.29,1.49)	**0.26 (0.13,0.52)**
THR (NEC)	**2.51 (1.93,3.25)**	*	1.26 (0.29,5.42)	*
TKR (cemented)	**1.94 (1.76,2.15)**	0.31 (0.05,2.13)	0.90 (0.41,1.96)	**0.24 (0.12,0.51)**
TKR (no cement)	**2.14 (1.79,2.54)**	*	2.06 (0.78,5.41)	*
TKR (NEC)	**2.08 (1.83,2.36)**	0.18 (0.02,1.60)	1.71 (0.57,5.17)	**0.22 (0.09,0.54)**
THR (cemented acetabulum)	**1.84 (1.56,2.17)**	*	0.68 (0.07,6.72)	1.44 (0.30,6.84)
THR (cemented femoral stem)	**1.91 (1.69,2.16)**	0.21 (0.03,1.30)	0.58 (0.20,1.72)	**0.04 (0.01,0.19)**
