# Supplementary material for: Outcomes for surgical procedures funded by the English health service but carried out in public versus independent hospitals: a database study
Source: BMJ Qual Saf. 2021 Sep 7;31(7):515–25. doi: 10.1136/bmjqs-2021-013522 (PMC9234423; doi:10.1136/bmjqs-2021-013522)
Supplement: Supplementary data [file bmjqs-2021-013522supp016.pdf]

Supplementary Table 11: Hazard ratios for all outcomes post-discharge and within 28 days (readmission, death), comparing NHS hospitals and for-profit ISHPs. Results highlighted in bold are significant at the 95% level. The * indicates hazard ratios that could not be reliably estimated because there were zero events for one or both of the provider types.

	Hazard ratio (95% CI) for for-profit ISHPs vs NHS hospitals		
**Operation**	**Within specialty readmission**	**All-cause readmission**	**Death**
Wisdom tooth impacted	**0.44 (0.26,0.74)**	**0.62 (0.45,0.87)**	*
Wisdom tooth NEC	**0.37 (0.20,0.67)**	0.72 (0.50,1.02)	*
Cholecystectomy	**0.72 (0.64,0.80)**	**0.71 (0.64,0.78)**	*
Prostate resection	**0.47 (0.35,0.63)**	**0.56 (0.48,0.65)**	0.75 (0.26,2.15)
Hysterectomy	**0.58 (0.50,0.66)**	**0.65 (0.58,0.73)**	1.00 (0.06,15.37)
IH repair (prosthetics)	**0.44 (0.35,0.55)**	**0.54 (0.46,0.65)**	1.25 (0.34,4.53)
UH repair (prosthetics)	**0.41 (0.33,0.52)**	**0.45 (0.38,0.53)**	0.50 (0.09,2.63)
UH repair (sutures)	**0.44 (0.34,0.57)**	**0.51 (0.42,0.62)**	0.33 (0.04,3.16)
VH repair (prosthetics)	**0.32 (0.25,0.42)**	**0.34 (0.28,0.42)**	1.00 (0.14,7.05)
Lumbar decompression	**0.40 (0.30,0.52)**	**0.55 (0.47,0.66)**	1.00 (0.17,5.86)
THR (cemented)	0.84 (0.71,1.00)	0.90 (0.80,1.02)	1.04 (0.52,2.11)
THR (no cement)	**0.80 (0.64,1.00)**	**0.74 (0.66,0.84)**	0.99 (0.37,2.64)
THR (NEC)	**0.53 (0.33,0.85)**	**0.66 (0.51,0.84)**	0.97 (0.13,7.15)
TKR (cemented)	**0.53 (0.44,0.65)**	**0.73 (0.67,0.81)**	1.16 (0.53,2.53)
TKR (no cement)	**0.38 (0.25,0.57)**	**0.70 (0.57,0.86)**	1.49 (0.26,8.59)
TKR (NEC)	**0.48 (0.35,0.64)**	**0.73 (0.62,0.85)**	2.13 (0.76,5.92)
THR (cemented acetabulum)	**0.55 (0.34,0.90)**	**0.64 (0.47,0.87)**	1.49 (0.29,7.72)
THR (cemented femoral stem)	**0.67 (0.53,0.84)**	**0.76 (0.66,0.87)**	1.54 (0.65,3.67)
