# Supplementary material for: Outcomes for surgical procedures funded by the English health service but carried out in public versus independent hospitals: a database study
Source: BMJ Qual Saf. 2021 Sep 7;31(7):515–25. doi: 10.1136/bmjqs-2021-013522 (PMC9234423; doi:10.1136/bmjqs-2021-013522)

Supplementary Figure 5: Cumulative incidence of discharge and readmission split by provider type and stratified by the time window in which the operation took place. The early and late time periods correspond to pre- and post- 1st Jan 2014 respectively. Cumulative incidence of discharge is shown in the first and third columns while readmission is shown in the second and fourth columns.

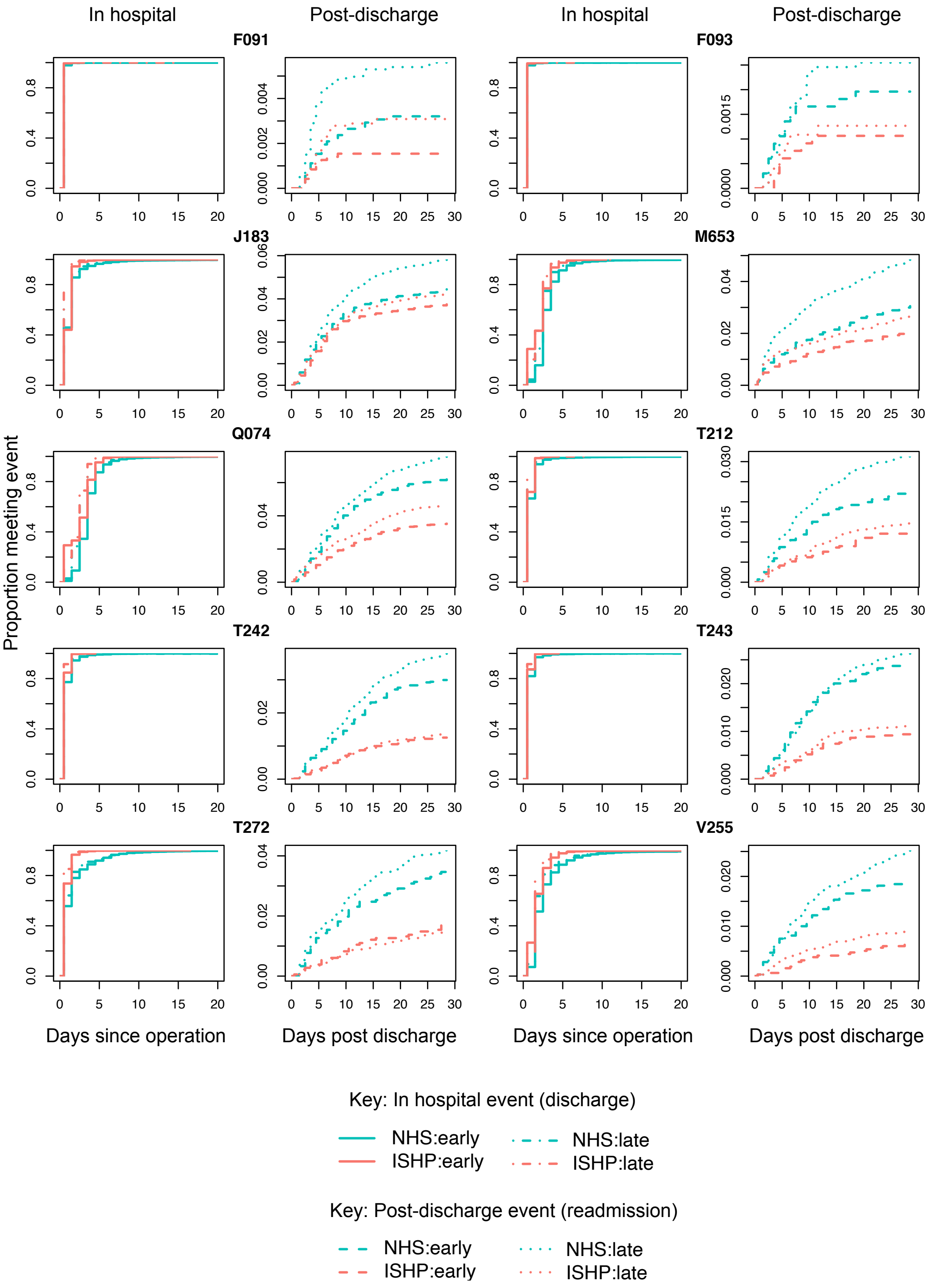

Supplementary Figure 5 (cont.)

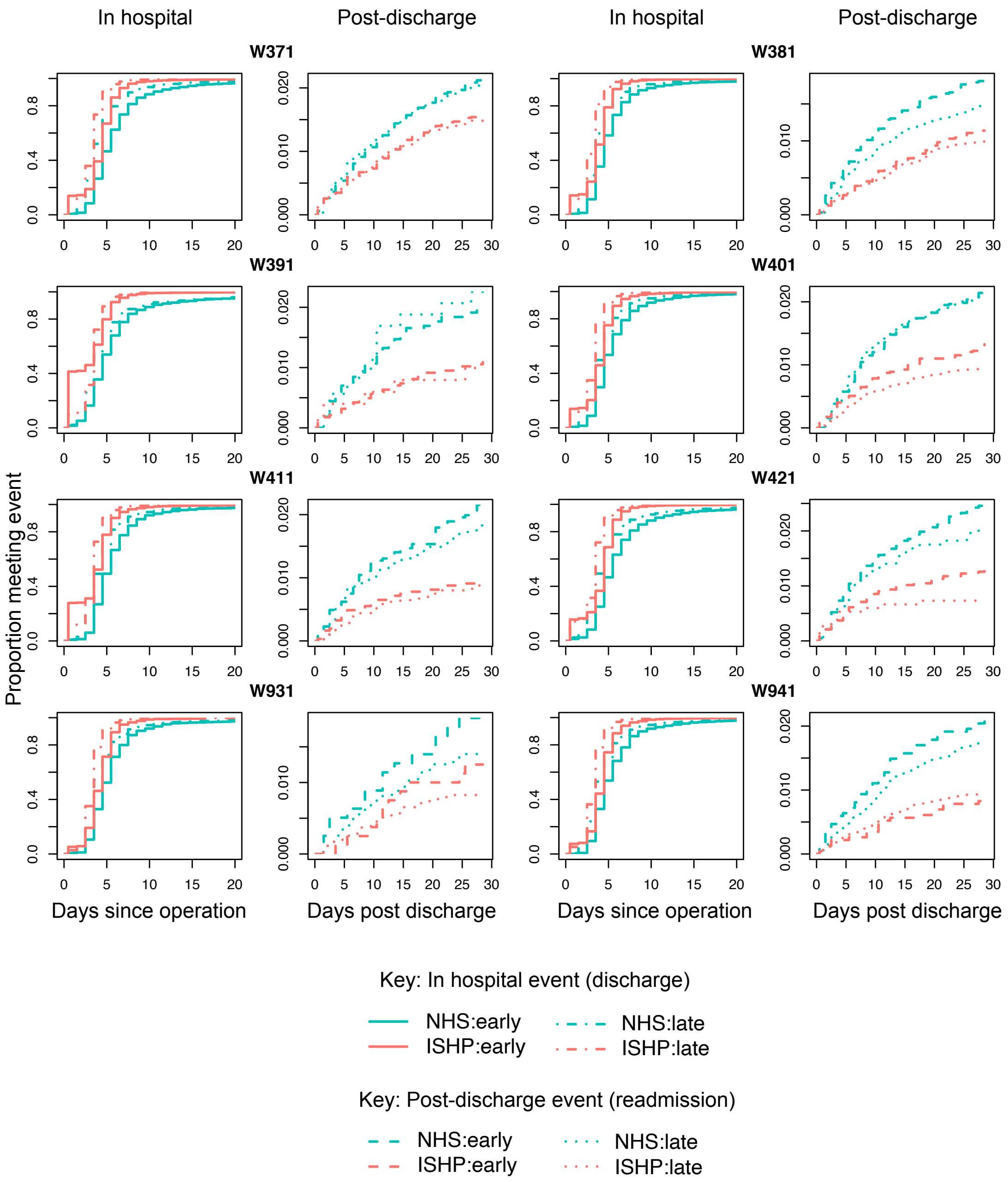

Supplement: Supplementary data [file bmjqs-2021-013522supp017.pdf]
