# Supplementary material for: Outcomes for surgical procedures funded by the English health service but carried out in public versus independent hospitals: a database study
Source: BMJ Qual Saf. 2021 Sep 7;31(7):515–25. doi: 10.1136/bmjqs-2021-013522 (PMC9234423; doi:10.1136/bmjqs-2021-013522)

Supplementary Figure 6: Hazard ratio estimates and 95% CIs for operations in ISHPs vs NHS hospitals, split by propensity score quartile. A hazard greater than 1 indicates that the event is more likely at ISHPs. *s situated at the top right of each figure indicate the size of the p-value supporting the inclusion of the interaction between provider type and propensity score in the model (* = $p < 0.05$; ** = $p < 0.01$; *** = $p > 0.001$). Readmission results relate to within-specialty readmissions. Recall that the “higher propensity” group are more likely to be treated in an ISHP and are on average more affluent, more likely to be ethnically white, and have a lower comorbidity score than the “lower propensity” group (Supplementary Table 5). Umbilical hernia repair using insert of prosthetic material (T242) is an example of an operation type for which the effect of provider type on discharge was smaller in magnitude for the higher propensity groups. The opposite pattern is seen for total prosthetic replacement of hip joint NEC (W391), where the hazard ratio for discharge was largest for the highest propensity quartile than for the other quartiles.

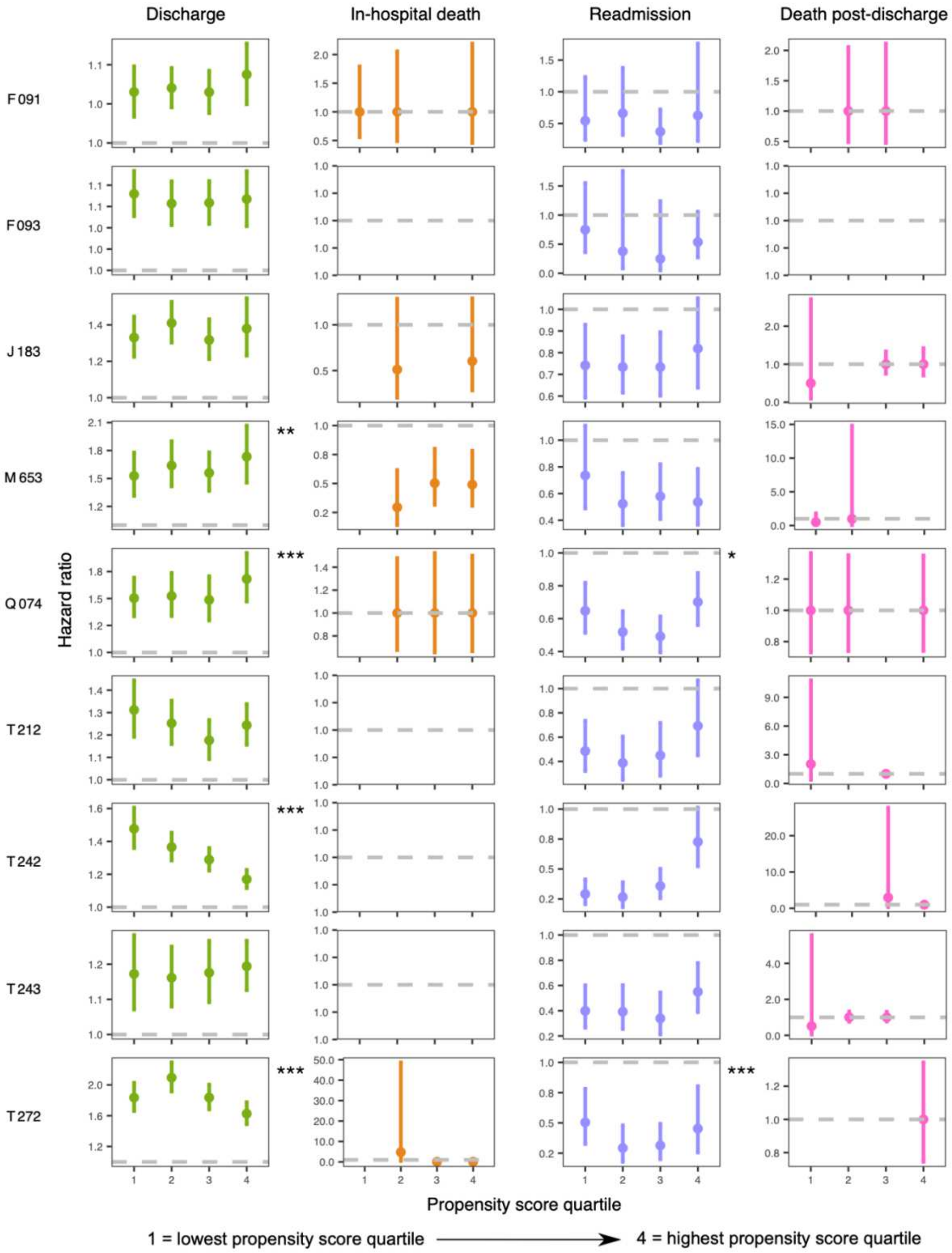
Discharge
In-hospital death
Readmission
Death post-discharge
F 091
F 093
J 183
M 653
Q 074
T 212
T 242
T 243
T 272
Hazard ratio
Propensity score quartile
1 = lowest propensity score quartile
4 = highest propensity score quartile

Supplementary Figure 6 (cont).

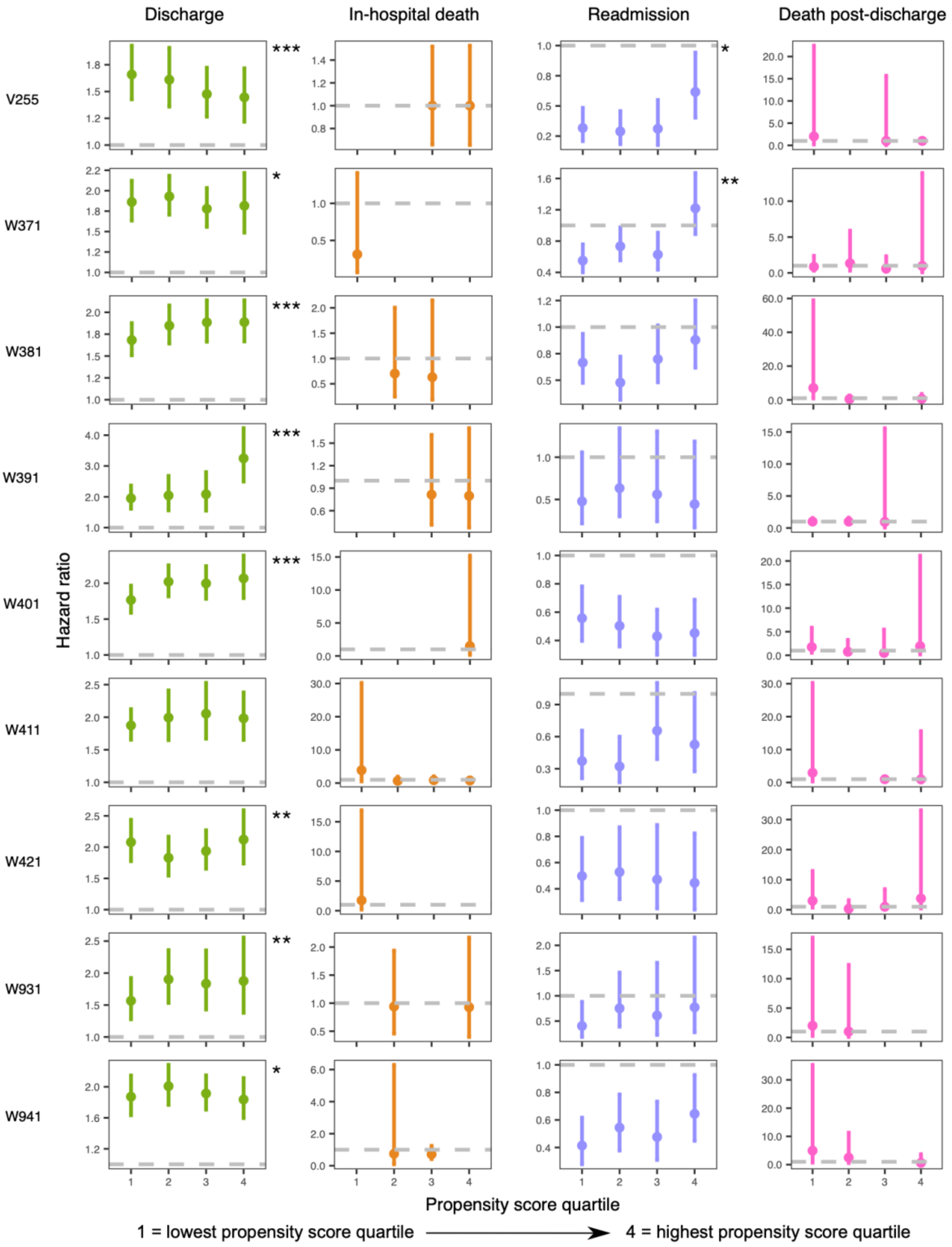

Supplement: Supplementary data [file bmjqs-2021-013522supp018.pdf]
