# Supplementary material for: Outcomes for surgical procedures funded by the English health service but carried out in public versus independent hospitals: a database study
Source: BMJ Qual Saf. 2021 Sep 7;31(7):515–25. doi: 10.1136/bmjqs-2021-013522 (PMC9234423; doi:10.1136/bmjqs-2021-013522)
Supplement: Supplementary data [file bmjqs-2021-013522supp019.pdf]

Supplementary Table 12: Hazard ratios for all in-hospital outcomes (discharge, in-hospital death, between-hospital transfer), when matching was done using Elixhauser comorbidity categories. Results highlighted in bold are significant at the 95% level. The * indicates hazard ratios that could not be reliably estimated because there were zero events for one or both of the provider types.

	Hazard ratio (95% CI) for ISHP vs NHS hospital			
**Operation**	**Discharge**	**Death**	**Emergency transfer**	**Other transfer**
Wisdom tooth impacted	**1.06 (1.04,1.08)**	*	*	*
Wisdom tooth NEC	**1.08 (1.06,1.10)**	*	*	0.12 (0.01,1.31)
Cholecystectomy	**1.31 (1.21,1.41)**	*	3.32 (0.92,11.92)	0.73 (0.23,2.33)
Prostate resection	**1.55 (1.36,1.77)**	*	**4.40 (1.06,18.20)**	0.71 (0.13,3.81)
Hysterectomy	**1.50 (1.33,1.69)**	*	1.89 (0.56,6.37)	0.75 (0.21,2.74)
IH repair (prosthetics)	**1.26 (1.18,1.34)**	*	0.79 (0.02,26.54)	0.55 (0.05,5.82)
UH repair (prosthetics)	**1.33 (1.26,1.40)**	*	1.11 (0.19,6.58)	0.18 (0.02,1.47)
UH repair (sutures)	**1.16 (1.09,1.23)**	*	*	0.86 (0.07,9.96)
VH repair (prosthetics)	**1.79 (1.66,1.92)**	*	1.15 (0.07,20.12)	1.00 (0.06,15.96)
Lumbar decompression	**1.51 (1.32,1.73)**	2.60 (0.36,18.80)	1.22 (0.41,3.58)	**0.21 (0.06,0.72)**
THR (cemented)	**1.73 (1.53,1.96)**	0.28 (0.07,1.17)	**2.50 (1.32,4.75)**	**0.33 (0.20,0.54)**
THR (no cement)	**1.73 (1.55,1.93)**	*	0.86 (0.36,2.07)	**0.32 (0.16,0.65)**
THR (NEC)	**2.03 (1.63,2.54)**	*	0.98 (0.30,3.18)	*
TKR (cemented)	**1.85 (1.68,2.03)**	0.24 (0.03,1.82)	**2.54 (1.20,5.38)**	**0.33 (0.17,0.65)**
TKR (no cement)	**1.88 (1.60,2.20)**	0.56 (0.08,4.06)	**3.74 (1.67,8.37)**	**0.15 (0.05,0.43)**
TKR (NEC)	**1.94 (1.68,2.23)**	0.21 (0.02,1.80)	3.13 (1.00,9.82)	**0.19 (0.09,0.40)**
THR (cemented acetabulum)	**1.73 (1.38,2.17)**	*	1.76 (0.37,8.41)	1.26 (0.46,3.41)
THR (cemented femoral stem)	**1.77 (1.59,1.97)**	0.25 (0.04,1.64)	2.35 (0.99,5.58)	**0.34 (0.15,0.78)**
