# Supplementary material for: Outcomes for surgical procedures funded by the English health service but carried out in public versus independent hospitals: a database study
Source: BMJ Qual Saf. 2021 Sep 7;31(7):515–25. doi: 10.1136/bmjqs-2021-013522 (PMC9234423; doi:10.1136/bmjqs-2021-013522)
Supplement: Supplementary data [file bmjqs-2021-013522supp020.pdf]

Supplementary Table 13: Hazard ratios for all outcomes post-discharge and within 28 days (readmission, death), when matching was done using Elixhauser comorbidity categories. Results highlighted in bold are significant at the 95% level. The * indicates hazard ratios that could not be reliably estimated because there were zero events for one or both of the provider types.

	Hazard ratio (95% CI) for ISHP vs NHS hospital		
**Operation**	**Within specialty readmission**	**All cause readmission**	**Death**
Wisdom tooth impacted	**0.50 (0.29,0.85)**	**0.64 (0.46,0.91)**	*
Wisdom tooth NEC	**0.42 (0.23,0.77)**	**0.70 (0.51,0.95)**	*
Cholecystectomy	**0.81 (0.72,0.92)**	**0.81 (0.73,0.89)**	1.67 (0.41,6.77)
Prostate resection	**0.52 (0.40,0.68)**	**0.60 (0.53,0.69)**	0.56 (0.17,1.82)
Hysterectomy	**0.58 (0.50,0.66)**	**0.66 (0.59,0.74)**	*
IH repair (prosthetics)	**0.46 (0.37,0.57)**	**0.57 (0.48,0.67)**	1.25 (0.35,4.50)
UH repair (prosthetics)	**0.37 (0.30,0.46)**	**0.43 (0.36,0.50)**	1.33 (0.31,5.71)
UH repair (sutures)	**0.40 (0.32,0.52)**	**0.49 (0.41,0.58)**	2.00 (0.18,22.00)
VH repair (prosthetics)	**0.36 (0.28,0.46)**	**0.40 (0.33,0.48)**	0.67 (0.11,3.95)
Lumbar decompression	**0.42 (0.33,0.54)**	**0.56 (0.48,0.66)**	1.33 (0.31,5.63)
THR (cemented)	0.93 (0.78,1.12)	0.90 (0.79,1.01)	1.20 (0.59,2.47)
THR (no cement)	**0.65 (0.52,0.81)**	**0.69 (0.61,0.79)**	1.59 (0.73,3.46)
THR (NEC)	**0.55 (0.37,0.83)**	**0.66 (0.52,0.83)**	*
TKR (cemented)	**0.49 (0.40,0.59)**	**0.77 (0.69,0.86)**	1.08 (0.50,2.32)
TKR (no cement)	**0.44 (0.32,0.60)**	**0.71 (0.60,0.84)**	1.32 (0.28,6.21)
TKR (NEC)	**0.54 (0.41,0.72)**	**0.74 (0.61,0.90)**	**3.68 (1.28,10.54)**
THR (cemented acetabulum)	0.71 (0.43,1.18)	**0.69 (0.52,0.91)**	5.98 (0.94,38.11)
THR (cemented femoral stem)	**0.57 (0.45,0.72)**	**0.78 (0.69,0.89)**	1.49 (0.62,3.60)
