# Supplementary material for: Outcomes for surgical procedures funded by the English health service but carried out in public versus independent hospitals: a database study
Source: BMJ Qual Saf. 2021 Sep 7;31(7):515–25. doi: 10.1136/bmjqs-2021-013522 (PMC9234423; doi:10.1136/bmjqs-2021-013522)
Supplement: Supplementary data [file bmjqs-2021-013522supp021.pdf]

Supplementary Table 14: Hazard ratios for all in-hospital outcomes (discharge, in-hospital death, between-hospital transfer), for Cox models run on raw (unmatched) data with adjustment. Results highlighted in bold are significant at the 95% level. The * indicates hazard ratios that could not be reliably estimated because there were zero events for one or both of the provider types.

	Hazard ratio (95% CI) for ISHP vs NHS hospital			
**Operation**	**Discharge**	**Death**	**Emergency transfer**	**Other transfer**
Wisdom tooth impacted	**1.06 (1.04, 1.08)**	3.32 (0.26, 41.95)	*	*
Wisdom tooth NEC	**1.07 (1.06, 1.08)**	*	1.57 (0.46, 5.38)	0.10 (0.01, 1.06)
Cholecystectomy	**1.38 (1.28, 1.48)**	*	**2.06 (1.17, 3.65)**	0.66 (0.36, 1.22)
Prostate resection	**1.64 (1.42, 1.88)**	*	**5.26 (1.86, 14.84)**	0.55 (0.14, 2.26)
Hysterectomy	**1.63 (1.43, 1.85)**	*	2.82 (0.91, 8.78)	0.81 (0.27, 2.43)
IH repair (prosthetics)	**1.25 (1.18, 1.33)**	*	0.68 (0.07, 6.28)	0.32 (0.04, 2.61)
UH repair (prosthetics)	**1.32 (1.26, 1.38)**	*	2.16 (0.47, 9.86)	0.17 (0.02, 1.37)
UH repair (sutures)	**1.14 (1.08, 1.22)**	*	*	0.24 (0.03, 1.90)
VH repair (prosthetics)	**1.86 (1.74, 1.99)**	**6.49 (1.17, 35.98)**	2.55 (0.25, 26.51)	0.71 (0.10, 4.79)
Lumbar decompression	**1.58 (1.36, 1.83)**	1.41 (0.21, 9.57)	1.31 (0.49, 3.47)	**0.20 (0.06, 0.65)**
THR (cemented)	**2.01 (1.79, 2.25)**	**0.15 (0.06, 0.40)**	**2.14 (1.21, 3.79)**	**0.26 (0.16, 0.42)**
THR (no cement)	**1.93 (1.72, 2.16)**	**0.17 (0.04, 0.72)**	1.39 (0.76, 2.57)	**0.32 (0.18, 0.57)**
THR (NEC)	**2.43 (1.93, 3.06)**	*	1.32 (0.47, 3.73)	*
TKR (cemented)	**2.13 (1.93, 2.34)**	**0.19 (0.09, 0.38)**	**1.68 (1.03, 2.73)**	**0.32 (0.21, 0.50)**
TKR (no cement)	**2.17 (1.89, 2.49)**	0.30 (0.04, 2.64)	**3.20 (1.58, 6.51)**	**0.16 (0.06, 0.41)**
TKR (NEC)	**2.11 (1.87, 2.38)**	0.16 (0.02, 1.28)	**2.45 (1.29, 4.67)**	**0.20 (0.09, 0.45)**
THR (cemented acetabulum)	**1.99 (1.60, 2.49)**	*	1.29 (0.27, 6.18)	1.09 (0.38, 3.14)
THR (cemented femoral stem)	**2.06 (1.84, 2.31)**	0.16 (0.03, 1.02)	1.92 (0.96, 3.84)	**0.30 (0.13, 0.70)**
