# Supplementary material for: Outcomes for surgical procedures funded by the English health service but carried out in public versus independent hospitals: a database study
Source: BMJ Qual Saf. 2021 Sep 7;31(7):515–25. doi: 10.1136/bmjqs-2021-013522 (PMC9234423; doi:10.1136/bmjqs-2021-013522)
Supplement: Supplementary data [file bmjqs-2021-013522supp022.pdf]

Supplementary Table 15: Hazard ratios for all outcomes post-discharge and within 28 days (readmission, death), for Cox models run on raw (unmatched) data with adjustment. Results highlighted in bold are significant at the 95% level. The * indicates hazard ratios that could not be reliably estimated because there were zero events for one or both of the provider types.

	**Hazard ratio (95% CI) for ISHP vs NHS hospital**		
**Operation**	**Within specialty readmission**	**All cause readmission**	**Death**
Wisdom tooth impacted	**0.50 (0.31, 0.81)**	**0.66 (0.48, 0.90)**	*
Wisdom tooth NEC	**0.41 (0.23, 0.75)**	**0.72 (0.56, 0.92)**	*
Cholecystectomy	**0.72 (0.65, 0.79)**	**0.72 (0.66, 0.78)**	1.03 (0.56, 1.89)
Prostate resection	**0.53 (0.41, 0.69)**	**0.61 (0.54, 0.69)**	0.80 (0.35, 1.84)
Hysterectomy	**0.60 (0.53, 0.68)**	**0.69 (0.62, 0.76)**	0.43 (0.06, 2.96)
IH repair (prosthetics)	**0.47 (0.39, 0.57)**	**0.59 (0.52, 0.68)**	1.57 (0.60, 4.10)
UH repair (prosthetics)	**0.37 (0.30, 0.46)**	**0.43 (0.37, 0.50)**	0.97 (0.35, 2.72)
UH repair (sutures)	**0.42 (0.33, 0.54)**	**0.49 (0.42, 0.59)**	0.84 (0.20, 3.57)
VH repair (prosthetics)	**0.34 (0.27, 0.43)**	**0.37 (0.32, 0.44)**	1.12 (0.27, 4.65)
Lumbar decompression	**0.38 (0.30, 0.48)**	**0.54 (0.47, 0.62)**	1.29 (0.45, 3.75)
THR (cemented)	**0.79 (0.70, 0.89)**	**0.82 (0.76, 0.89)**	0.98 (0.67, 1.43)
THR (no cement)	**0.65 (0.55, 0.77)**	**0.70 (0.65, 0.77)**	1.21 (0.75, 1.95)
THR (NEC)	**0.58 (0.41, 0.81)**	**0.65 (0.53, 0.79)**	0.85 (0.23, 3.19)
TKR (cemented)	**0.50 (0.45, 0.56)**	**0.72 (0.68, 0.77)**	1.16 (0.91, 1.49)
TKR (no cement)	**0.43 (0.32, 0.58)**	**0.69 (0.58, 0.82)**	1.33 (0.36, 4.98)
TKR (NEC)	**0.50 (0.40, 0.63)**	**0.71 (0.61, 0.83)**	**2.97 (1.36, 6.48)**
THR (cemented acetabulum)	**0.55 (0.35, 0.85)**	**0.64 (0.51, 0.80)**	**4.71 (1.75, 12.72)**
THR (cemented femoral stem)	**0.59 (0.49, 0.71)**	**0.75 (0.67, 0.84)**	1.36 (0.74, 2.48)
